# Ideal Bi-Based Hybrid Anode Material for Ultrafast Charging of Sodium-Ion Batteries at Extremely Low Temperatures

**DOI:** 10.1007/s40820-024-01560-9

**Published:** 2024-11-13

**Authors:** Jie Bai, Jian Hui Jia, Yu Wang, Chun Cheng Yang, Qing Jiang

**Affiliations:** Key Laboratory of Automobile Materials (Jilin University), Ministry of Education, School of Materials Science and Engineering, Jilin University, Changchun, 130022 People’s Republic of China

**Keywords:** Bi nanoparticles, High temperature shock, High-rate activation, Ultrafast charging, Low-temperature sodium-ion batteries

## Abstract

Sodium-ion batteries have emerged as competitive substitutes for low-temperature applications due to severe capacity loss and safety concerns of lithium-ion batteries at − 20 °C or lower. However, the key capability of ultrafast charging at ultralow temperature for SIBs is rarely reported. Herein, a hybrid of Bi nanoparticles embedded in carbon nanorods is demonstrated as an ideal material to address this issue, which is synthesized via a high temperature shock method. Such a hybrid shows an unprecedented rate performance (237.9 mAh g^−1^ at 2 A g^−1^) at − 60 °C, outperforming all reported SIB anode materials. Coupled with a Na_3_V_2_(PO_4_)_3_ cathode, the energy density of the full cell can reach to 181.9 Wh kg^−1^ at − 40 °C. Based on this work, a novel strategy of high-rate activation is proposed to enhance performances of Bi-based materials in cryogenic conditions by creating new active sites for interfacial reaction under large current.

**Supplementary Information:**

The online version contains supplementary material available at 10.1007/s40820-024-01560-9.

## Highlights

- Metallic nanoparticles with excellent size controllability and high loading rate are obtained via ultrafast high temperature shock method.
- The Bi/CNRs-15 electrode exhibits an unprecedented rate performance (237.9 mAh g^−1^ at 2 A g^−1^) at − 60 °C, while the energy density of the full cell can reach to 181.9 Wh kg^−1^ at − 40 °C.
- A novel strategy of high-rate activation is proposed to obtain high capacity and superior stability at low temperature by creating new active sites for interfacial reaction.

**Supplementary Information:**

The online version contains supplementary material available at 10.1007/s40820-024-01560-9.

## Graphical abstract

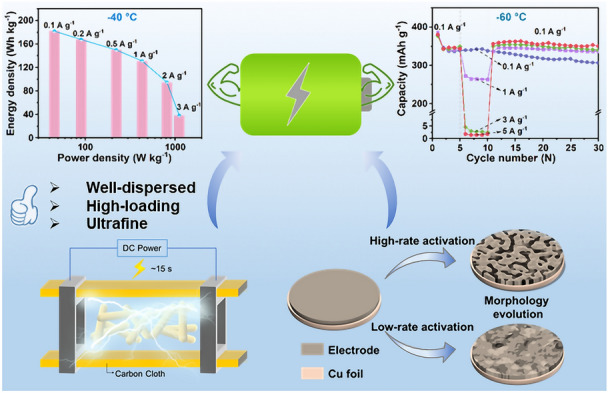

## Introduction

Lithium-ion batteries (LIBs) have accounted for a dominant position in energy storage fields from portable electronic equipment to electric vehicles (EVs) thanks to their high energy density and long lifespan [1–3]. However, severe ionic conductivity decline, sluggish interfacial charge transfer kinetics, fatal dendrite formation and fragile electrolyte/electrode interphase may all deal a crushing blow to the battery performances and safety concerns at subzero temperature, seriously restricting practical applications at high latitudes or altitudes localities, and the fields of civil and military [4–6]. As a consequence, developing advanced energy storage devices with superior temperature tolerance, which could accommodate to the harsh working conditions brought by diverse seasons and regions, is highly desirable. Sodium-ion batteries (SIBs) have gained extensive concerns owing to wide distribution, cost-effectiveness and natural abundance of sodium resources [7–10]. Additionally, Na^+^ has smaller de-solvation energy compared with Li^+^, demonstrating a lower activation barrier for Na^+^ insertion/extraction and thus better electrochemical performances at low temperature [11–13]. Nonetheless, the low operating temperature, especially at high charging rates, could bring decreased ionic conductivity due to increased electrolyte viscosity and unstable interfacial reaction, resulting in severe polarization and capacity decay [14, 15]. So far, the studies of low-temperature SIBs principally focus on modulating the solvation structure and developing electrolytes with low freezing point, which is far from sufficient, let alone the ultrafast charging capability at ultralow temperature [16–18]. In order to promote practical applications of SIBs in cryogenic environments, it is also crucial to enhance the electrode electrochemical performance through structural modification, coating and other strategies [19, 20].

Alloy-type materials, featuring relatively low redox reaction potential and high energy density, have been considered to be promising anode candidates for SIBs [21–23]. Among them, Bi with large lattice spacing [d_(003)_ = 3.95 Å], high electronic conductivity (7.8 × 10^5^ S m^−1^) and theoretical capacity (385 mAh g^−1^), has attracted great attentions [24, 25]. Wang and coworkers reported that the pure Bi electrode combined with ether-based electrolytes exhibited prospective low-temperature behavior (370 mAh g^−1^ at 0.02 A g^−1^ at − 40 °C) owing to the solvent co-intercalation process [26]. Notwithstanding, the cycling stability and rate performance are still limited at ultralow temperature [27, 28]. Batteries with extremely fast charging capability and long life spans are highly expected on account of the demands for high-power EVs and tools in cold region [29]. It has been proved that designing unique nanostructures is an effective strategy to improve the rate property and cyclability [6, 30, 31]. Thermal treatment is frequently used to fabricate nanomaterials from metal salt precursors. However, the conventional methods based on tube furnace annealing (TFA) generally require relatively long synthesis duration and suffer from low energy utilization efficiency [32, 33]. Uneven heating conditions also give rise to nanoparticle aggregation. Therefore, a rapid and highly effective method for the synthesis of electrode materials is still in high demand. High temperature shock (HTS), a facile and ultrafast synthesis method, has been utilized not only in domains like catalysis, but also in the fabrication of battery electrode materials in recent years [34–37].

Herein, a hybrid of well-dispersed and high-loading Bi nanoparticles embedded in carbon nanorods is fabricated by ultrafast HTS for only 15 s (Bi/CNRs-15), which is demonstrated as an ideal material for ultralow-temperature ultrafast-charging SIBs. The instantaneously generated high temperature can effectively decompose the precursors of metal–organic frameworks (MOFs) to metallic Bi nanoparticles with excellent size controllability and high loading rate. The fast heating and cooling rates inhibit the aggregation of nanoparticles, which considerably shortens the ions/electrons diffusion path. As expected, the designed Bi/CNRs-15 anode displays incredible temperature tolerance, fast charging capability (261.4 mAh g^−1^ at a high rate of 5 A g^−1^) and cycling stability (241.7 mAh g^−1^ at 1 A g^−1^ after 2400 cycles) at − 40 °C. Even at an extremely low temperature of − 60 °C, the hybrid also delivers a high capacity of 237.9 mAh g^−1^ at 2 A g^−1^, surpassing all reported SIB anode materials. Furthermore, a stable and homogenous solid-electrolyte interface (SEI) layer with more inorganic species could improve structural stability and boost rate kinetics in some degree, which is unraveled by X-ray photoelectron spectroscopy etching. Moreover, it is worth noting that an unusual phenomenon of capacity increment named “negative fading” takes place after high-rate cycling under low-temperature conditions, which provides a new route to enhance the low-temperature performances of SIBs.

## Experimental Section

### Materials

All chemicals of bismuth chloride (BiCl_3_, 99%, Macklin Reagent Co., Ltd.), potassium iodide (KI, 99%, Aladdin Reagent Co., Ltd.), acetic acid (CH_3_COOH, 99.5%, Macklin Reagent Co., Ltd.), trimesic acid (H_3_BTC, 99.9%, Aladdin Reagent Co., Ltd.), methanol (CH_3_OH, 99%, Beijing Chemical Works) and dimethylformamide (DMF, 99%, Aladdin Reagent Co., Ltd.) were used without further purification. The ultrapure water used in all experiments was with a specific resistance of 18.2 MΩ cm.

### Preparation of Bi/CNRs-15

First, Bi-MOFs were synthesized via a modified two-step solvothermal method. Typically, BiCl_3_ (0.5 mmol) and KI (0.5 mmol) were dispersed in acetic acid (50 mL) and deionized (DI) water (12.5 mL), respectively. After fully stirred, these two solutions were mixed to form the tender green suspension and the pH value of the mixture was adjusted to 6. After 30 min of stirring, the above dispersion was transferred into a Polytetrafluoroethylene lined stainless steel autoclave and kept at 160 °C for 2 h. Then BiOI nanosheets (BiOI NSs) were obtained after centrifugation and freeze drying. Subsequently, BiOI NSs (0.5 g) and H_3_BTC (0.9 g) were dissolved in the mixture of dimethylformamide (22.5 mL) and methanol (7.5 mL) and magnetically stirred for 30 min. Thereafter, the obtained solution was shifted to a 50 mL Teflon-lined autoclave and held at 120 °C for 3 h. The powders of Bi-MOFs were assembled by filtration, washing and drying. Finally, the carbon cloth loaded with Bi-MOFs was clipped into copper electrodes of DC power to acquire Bi/CNRs-15, which was passed a current of 10 A in the argon-filled glovebox for 15 s. Besides, contrast samples shocked for 5 and 30 s were also synthesized, which are denoted as Bi/CNRs-5 and Bi/CNRs-30, respectively. As a comparison, Bi/CNRs-TFA was synthesized by a conventional TFA treatment. The same Bi-MOF precursors were placed in a tube furnace annealed at 600 °C for 2 h in an Ar/H_2_ (with 5 vol% H_2_) atmosphere.

### Materials Characterization

X-ray diffraction (XRD) analysis was performed on an instrument with Cu Kα (Rigaku D/Max-2550). Transmission electron microscopy (TEM, JEM-2100F, JEOL) and field-emission scanning electron microscopy (FESEM, JSM-6700F, JEOL) were fulfilled to obtain the structure and morphology of all samples. Raman spectra were carried out by a micro-Raman spectrometer (Renishaw). X-ray photoelectron spectroscopy (XPS) data were collected at an ESCALAB 250Xi system. Thermogravimetric analysis (TGA) was performed in the air using an SDT Q600 instrument. The nitrogen adsorption and desorption (Micromeritics ASAP 2020 analyzer) was used to test the specific surface area and pore sizes. The carbon vacancies were measured by the electron paramagnetic resonance (EPR) technique on a Bruker EMX plusmachine. *In-situ* XRD was performed on a Rigaku Smart lab. Low-temperature electrochemical tests were conducted in the high and low temperature test chamber (BPH-060).

### Electrochemical Measurements

All CR2025-type coin cells were assembled in an argon-filled glove box by employing Na metal as the counter electrode, Whatman glass fiber (GF/C) as the separator, and 1 M NaPF_6_ in 1,2-dimethoxyethane (DME) as the electrolyte. The active materials (Bi/CNRs-15, Bi/CNRs-5, Bi/CNRs-30, Bi/CNRs-TFA, pure Bi) were mixed with Super P and sodium carboxymethylcellulose with a weight ratio of 7:2:1. The mixed slurry was uniformly pasted on Cu foil and dried in a vacuum oven at 70 °C. Additionally, the mass loadings of active materials were ranging from 0.6 to 4.2 mg cm^−2^. In this work, all capacities are evaluated by the whole weight of the Bi/CNRs-15 composite. The electrodes were tested 3 cycles at 1 A g^−1^ at room temperature before transferring to the high and low temperature test chamber. The cathode consists of Na_3_V_2_(PO_4_)_3_ (NVP), Super P and polyvinylidene fluoride in a mass ratio of 7:2:1. The full cell was assembled using NVP and Bi/CNRs-15 as the cathode and anode, respectively, with the same electrolyte and separator. Meanwhile, the voltage range was selected at 1.5–3.3 V for the full cell, and the anode was electrochemically activated for 3 cycles before it was used in the full cell. The galvanostatic charge/discharge and GITT tests were implemented using a LAND-CT2001A battery testing system in 0.01–1.5 V (*vs.* Na^+^/Na) at room temperature/low temperature. The pulse current, duration and relaxation time of the GITT tests are 50 mA g^−1^, 600 s, and 3600 s, respectively. CV curves and temperature-dependent EIS measurements with a frequency range of 100 kHz to 0.01 Hz were performed on an electrochemical workstation (Ivium-n-Stat).

## Results and Discussion

### Synthesis and Characterization

**Fig. 1:**
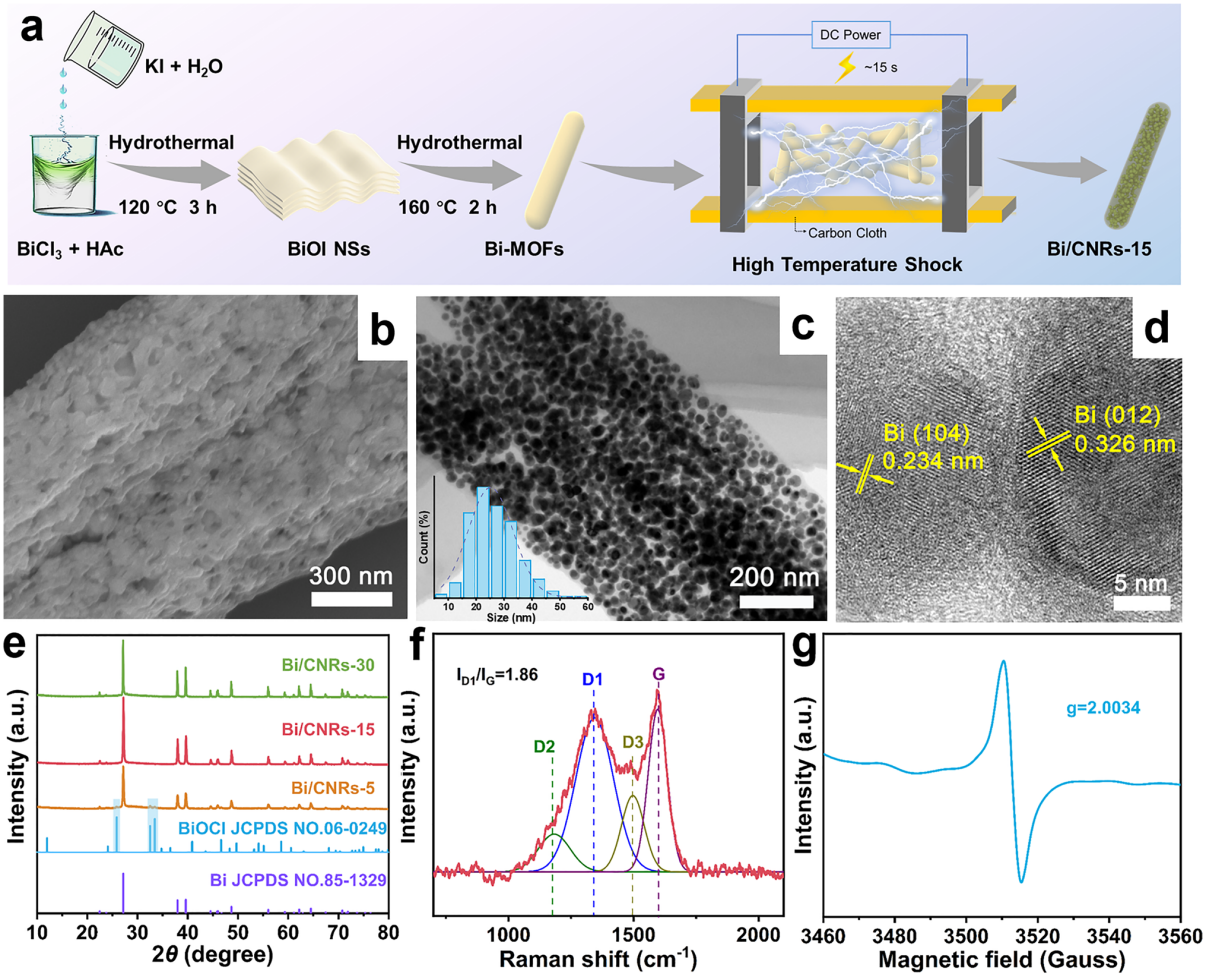
Schematic illustration and structural morphologies. **a** Schematic illustration of the synthesis route of Bi/CNRs-15. **b** SEM image of Bi/CNRs-15. **c** TEM image of Bi/CNRs-15. **d** HRTEM image of Bi/CNRs-15. **e** XRD patterns of Bi/CNRs-5, Bi/CNRs-15 and Bi/CNRs-30. **f** Raman spectrum of Bi/CNRs-15. **g** EPR spectrum of Bi/CNRs-15

The synthesis route of Bi/CNRs-15 is schematically illustrated in Fig. 1a. It has been reported that MOF-based materials present vast opportunities in the quest for optimal electrode materials for rechargeable batteries due to their high porosity, diverse structures and controllable chemical compositions [38–40]. Here, firstly, a Bi-MOF as the precursor (Fig. S1) was prepared by a modified two-step solvothermal reaction [41], in which bismuth chloride (BiCl_3_) as Bi precursor, and the ligand of trimesic acid (H_3_BTC) served as a structure-directing agent and carbon source [42]. Specifically, the carboxyl group (–COOH) of H_3_BTC lost its proton (H^+^) to form the carboxylate ion (–COO^−^) [43, 44]. Bi^3+^ acted as the central metal ion and coordinated with the -COO^−^ of the deprotonated H_3_BTC ligand to form a coordination bond [43, 45]. After HTS (Fig. S2) in Ar atmosphere for only 15 s, the final product of Bi/CNRs-15 was obtained, where Bi nanoparticles were *in-situ* formed from Bi-MOFs and uniformly embedded in the carbon nanorods. Meanwhile, under the extreme heating generated by HTS, the chlorine can effectively escape from BiOCl (hydrolyzed from BiCl_3_) through the reaction 2BiOCl + C = 2Bi + CO_2_ + Cl_2_ [46]. In order to optimize the synthesis condition, Bi/CNRs-5, Bi/CNRs-30 and Bi/CNRs-TFA were also prepared. All synthesis details are offered in the Experimental Section.

Figures S3 and 1b, c present the field-emission scanning electron microscopy (FESEM) and transmission electron microscopy (TEM) images of Bi/CNRs-15, respectively. Clearly, Bi nanoparticles with an average particle size of 26.5 nm (Fig. S4) are homogeneously loaded in the carbon nanorods, which profits from the rapid cooling/heating rate. In sharp contrast, the analogs of Bi/CNRs-TFA synthesized through a TFA method display aggregated particles, nonuniform size distribution and low metal loading (Figs. S5 and S6), which are principally caused by prolonged heat treatment and slow cooling/heating rate. The samples with different pulse time (5 and 30 s) are also presented in Fig. S7. It is evident that Bi/CNRs-15 exhibits the best dispersity in these three samples. Figure 1d shows the high-resolution TEM (HRTEM) image of Bi/CNRs-15, where highly crystallized nanoparticles can be observed with interlayer distances of 0.326 and 0.234 nm, corresponding to (012) and (104) planes of metallic Bi. The XRD patterns of Bi/CNRs-5, Bi/CNRs-15, Bi/CNRs-30, and Bi/CNRs-TFA are shown in Figs. 1e and S8, respectively. With increasing the thermal shock time, the XRD peaks assigned to BiOCl (JCPDS No. 06-0249) disappear until 15 s and the present sharp peaks are indexed to the high-crystallinity rhombohedral Bi phase (JCPDS No. 85-1329). Note that the average crystallite size of the Bi particles calculated from Scherrer equation (Table S1) is larger than that observed from TEM (26.5 nm). The possible reason is that XRD gives volume-weighted measurements, which tends to overestimate the geometric particle size [47–49]. Moreover, the well-defined crystallinity of Bi/CNRs-15 can be identified by the selected area electron diffraction (SAED) pattern in Fig. S9. A series of clear spotty diffraction rings portray the polycrystallinity of Bi/CNRs-15, further validating the XRD result [50]. As shown in Fig. S10, the bright-field scanning transmission electron microscopy (BF-STEM) and corresponding energy-dispersive spectrum (EDS) mapping images unambiguously reveal that Bi and C elements are homogeneously distributed in the Bi/CNRs-15 composite without obvious aggregation.

XPS was measured to investigate the surface characteristics of the Bi/CNRs-15 hybrid, where only C, Bi, and O elements are perceived (Fig. S11). The high-resolution C 1*s* spectrum (Fig. S12) can be deconvoluted into three peaks at 284.8, 285.7, and 289.2 eV, being ascribed to C–C/C=C, C–O, and C=O bonds, respectively [51]. Two pairs of peaks shown in the Bi 4f high-resolution XPS spectrum (Fig. S13) are assigned to metallic Bi (157.3 and 162.7 eV) and Bi_2_O_3_ (159.2 and 164.6 eV), resulting from unavoidable oxidation of highly active Bi in the nanoscale under ambient air [22, 27]. After etching 10 nm, the peak intensity of Bi_2_O_3_ (Fig. S13b) is obviously much weaker than that without etching, proving that Bi_2_O_3_ mainly exists on the surface of the sample. The high-resolution O 1*s* spectrum (Fig. S14) can be fitted into three peaks with the binding energy of 530.3, 532.4, and 533.9 eV, corresponding to Bi–O, C–O–Bi, and C–O bonds, respectively. The C–O–Bi bond is a chemical bond formed between C and O atoms, and the O atom also interacts with Bi, which involves the coordination of organic ligands to metal ion centers [44]. As observed in the Raman spectrum (Fig. 1f), there are two typical peaks located at 1340 and 1596 cm^−1^, attributing to D bands (defective structure) and G bands (graphite structure) of carbonous materials, respectively. Moreover, the D band can be further deconvoluted into three peaks at 1345 (D1), 1183 (D2), and 1497 (D3) cm^−1^, respectively. The D1 band corresponds to the disorders and defects in carbon lattice and the D2 band is ascribed to edge defects, while the D3 band is associated with amorphous *sp*^3^ carbon [52–54]. The calculated ratios of *I*_D1_/*I*_G_ based on integrated peak intensity is 1.86, disclosing abundant defects and disorders in Bi/CNRs-15. To further corroborate defects in Bi/CNRs-15, electron paramagnetic resonance (EPR) characterization was also carried out. As presented in Figs. 1g and S15, the strong EPR signal at *g* = 2.0034 is associated with the rich carbon vacancies in the Bi/CNRs-15 hybrid [53], which is well consistent with the Raman spectrum. Compared to Bi/CNRs-TFA, the continuous gas emission can sacrifice irreversible heteroatom defects while induce more reversible carbon vacancies under extreme heating conditions derived from HTS [54]. These abundant vacancies are able to furnish more active sites for the absorption and diffusion of Na^+^, which is beneficial for Na storage [55]. The Bi content in Bi/CNRs-15 is determined by the TGA in air, which is calculated to be 86.8% from the equation in Fig. S16. To further study the specific surface area and pore texture type of Bi-MOFs and Bi/CNRs-15, nitrogen adsorption–desorption technique was implemented. As shown in Fig. S17, Bi/CNRs-15 displays a typical mesoporous structure with a specific surface area of 48.1 m^2^ g^−1^. The high porosity increases the contact areas between the electrolyte and electrode, and decreases the Na^+^ diffusion distance, facilitating the rate performance of Bi/CNRs-15 [56].

### Low-Temperature Performance

**Fig. 2:**
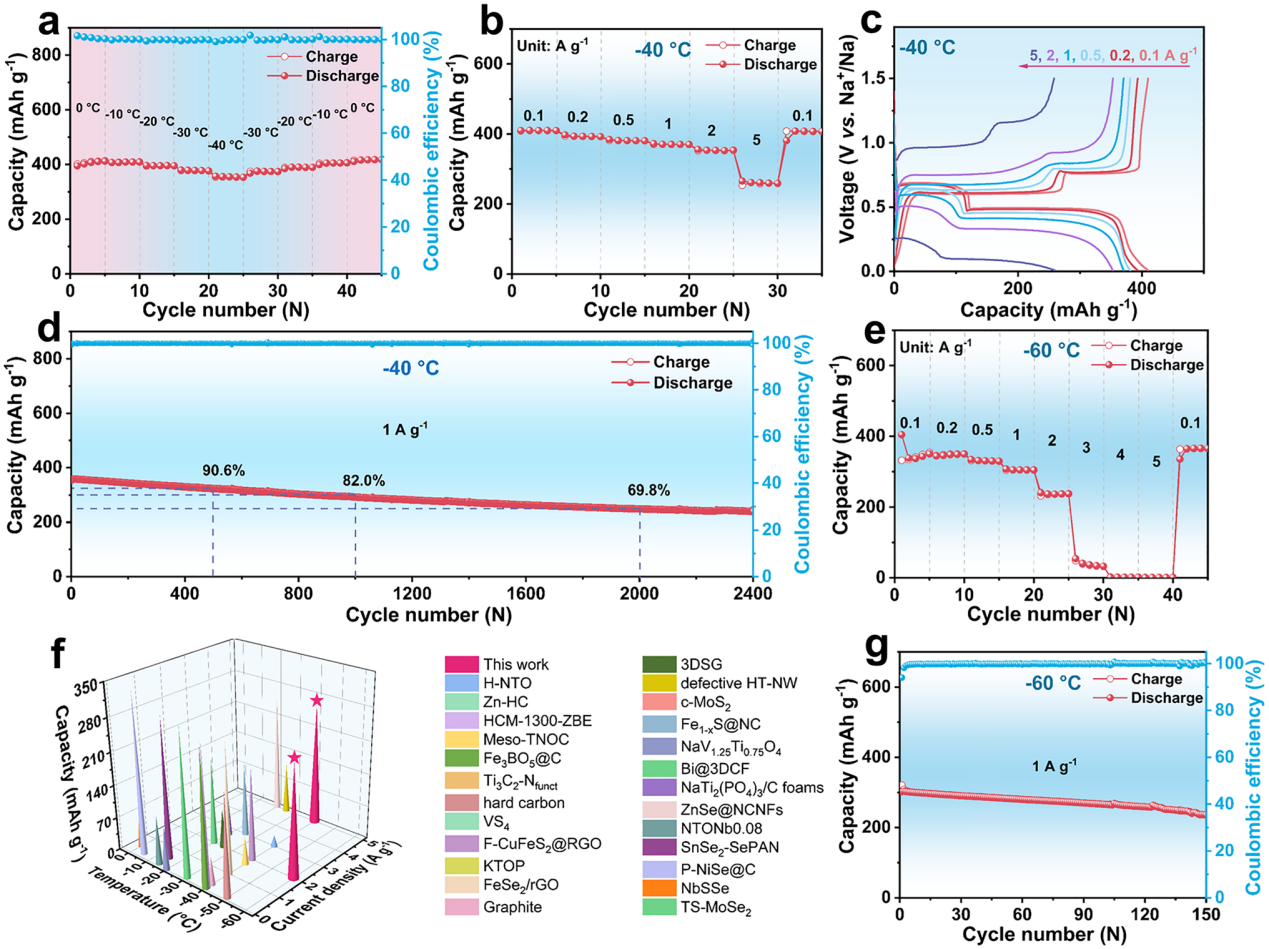
Half-cell performance at ultralow temperature. **a** Cycling performance of Bi/CNRs-15 at 1 A g^−1^ at different temperatures. **b** Rate performance of Bi/CNRs-15 at − 40 °C. **c** Charge/discharge curves of Bi/CNRs-15 at various rates at − 40 °C. **d** Long-term cycling performance of Bi/CNRs-15 at 1 A g^−1^ at − 40 °C. **e** Rate performance of Bi/CNRs-15 at − 60 °C. **f** Comparisons of rate performance with reported low-temperature SIB anodes. **g** Cycling performance of Bi/CNRs-15 at 1 A g^−1^ at − 60 °C

The Na storage properties of Bi/CNRs-15 were investigated in 2025 coin-type half cell and full cell at room temperature, which shows remarkable high-rate performance and appealing cycling stability even under high mass loadings (Figs. S18-S27, detailed description in Supporting Information). Based on the well-tailored Bi/CNRs-15 electrode, half cells were also assembled with metallic Na to evaluate the electrochemical performance at low temperature. As shown in Fig. 2a, the Bi/CNRs-15 electrode exhibits admirable adaptability for temperature. The capacity of Bi/CNRs-15 testing at 1 A g^−1^ slightly decreases as the temperature drops to − 40 °C, and the capacity can be retrieved and maintain stability when the temperature returns to 0 °C. The discharge/charge curves (Fig. S29) display polarized plateaus below − 20 °C due to rapidly increasing internal resistance. The CV curves (Fig. S30) under low-temperature conditions show great repetition between the first and second cycles, indicating excellent cycling stability and Na^+^ storage reversibility of the Bi/CNRs-15 electrode. From Fig. 2b–d and S31, the Bi/CNRs-15 electrode represents ultrahigh rate capability (261.4 mAh g^−1^ at 5 A g^−1^) and superior cycling performance (240.3 mAh g^−1^ at 1 A g^−1^ after 2400 cycles with a capacity retention of 67.3%) at − 40 °C, while Bi/CNRs-TFA only exhibits moderate rate property (101.5 mAh g^−1^ at 5 A g^−1^). In sharp contrast, the pure Bi electrode is unstable and displays battery invalidity after only several cycles at 1 A g^−1^ at − 40 °C (Fig. S32). As presented in Figs. 2e and S33, even at − 60 °C, Bi/CNRs-15 still displays incredible rate property (237.9 mAh g^−1^ at 2 A g^−1^), which is much better than other anode materials previously reported in open literatures (Fig. 2f and Table S2). Simultaneously, Bi/CNRs-15 also exhibits competitive cycling stability (237.1 mAh g^−1^ at 1 A g^−1^ after 150 cycles) [Fig. 2g]. Surprisingly, the phenomenon of exceeding theoretical capacity appears in Fig. 2b, which has been reported for different kinds of electrode materials (carbon, metal, metal oxide, metal sulfides, etc.) [57–59]. In this work, the extra capacity may come from interfacial charge storage [58]. Additionally, carbonaceous materials can store extra Na in pores and cavities [59].

**Fig. 3:**
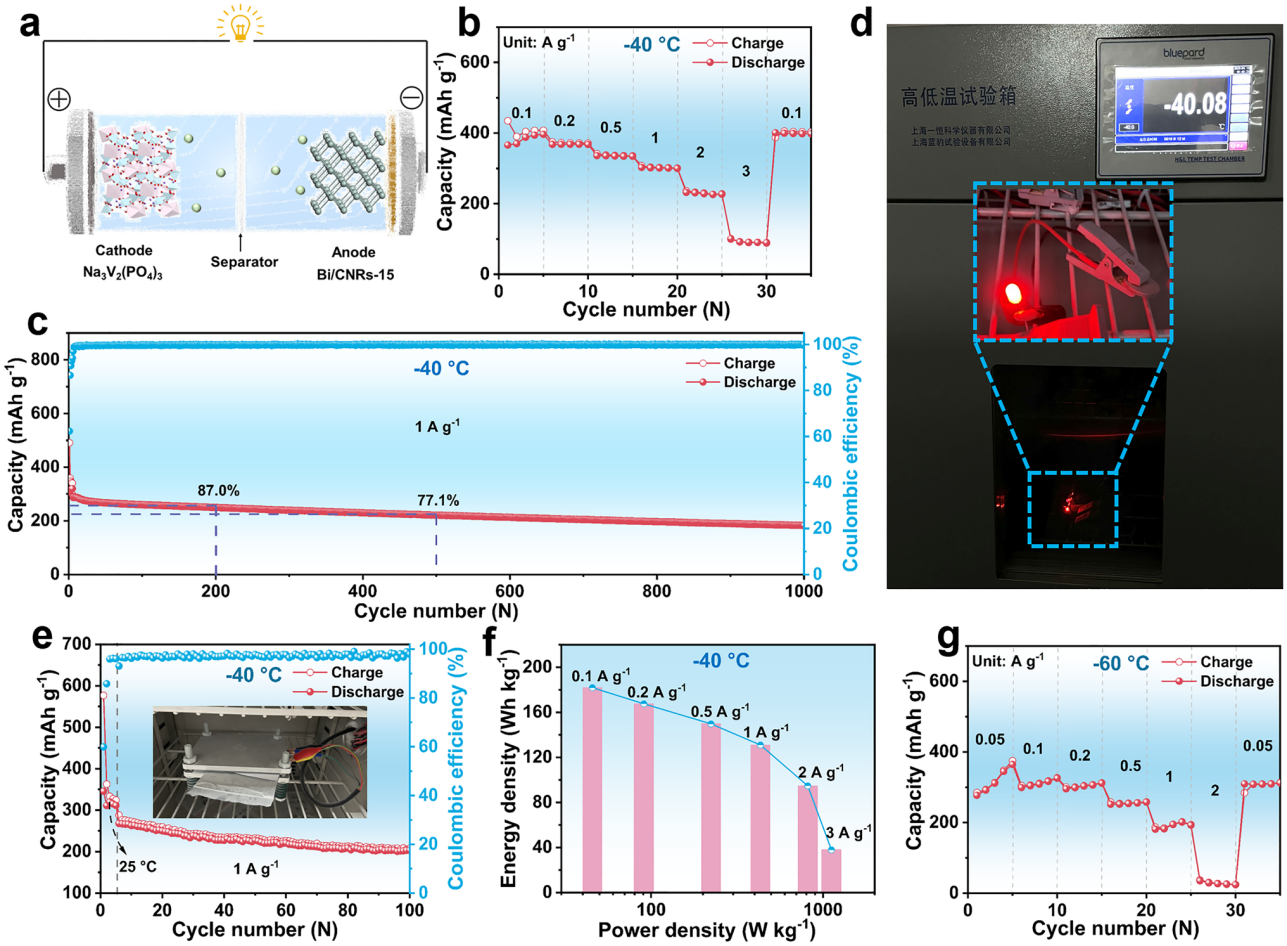
Full-cell performance at ultralow temperature. **a** The schematic diagram of the full cell with the Bi/CNRs-15 anode and NVP cathode (Bi/CNRs-15//NVP). **b** Rate performance of the Bi/CNRs-15//NVP full cell at − 40 °C. **c** Long-term cycling performance of the Bi/CNRs-15//NVP full cell at 1 A g^−1^ at − 40 °C. **d** The lighted LEDs driven by the Bi/CNRs-15//NVP full cell at − 40 °C. **e** Cycling performance of the Bi/CNRs-15//NVP pouch cell at 1 A g^−1^ at − 40 °C. **f** Ragone plot evaluated by the total mass of anode and cathode at − 40 °C. **g** Rate performance of the Bi/CNRs-15//NVP full cell at − 60 °C

To preliminarily estimate the practical applications of the Bi/CNRs-15 hybrid in extremely cold areas, the full cell was fabricated using Bi/CNRs-15 as an anode, and NVP as a cathode with a negative/positive (N/P) ratio of 1.01 (see Supporting Information for details) for the electrochemical performance test (Fig. 3a). The Bi/CNRs-15//NVP full cell exhibits excellent fast charging capability at − 40 °C with a high capacity of 382.9 mAh g^−1^ at 0.1 A g^−1^ (based on active mass of the anode), in which 58.9% of the capacity can be retained even when the current density increases by 20-folds from 0.1 to 2 A g^−1^ (Figs. 3b and S34). Note that the specific capacity of the full cell is lower than that of the half cell owing to the un-optimized cathode and the problem of matching the anode and cathode [60]. As illustrated in Fig. S35, the full cell displays appealing cycling stability (a capacity retention of 97.0% at 0.1 A g^−1^ after 100 cycles) after high-rate cycling. As shown in Fig. 3c, the full cell presents a stable capacity retention of 63.9% at 1 A g^−1^ over 1000 cycles. It should be noted that the charged full cell can power the light-emitting diodes (LEDs) at − 40 °C (Figs. 3d and S36). The practical feasibility is further evaluated by the pouch cell (Fig. 3e), which displays 75.8% capacity retention after 100 cycles at 1 A g^−1^. It should be noted that the coulombic efficiencies of the Bi/CNR-15//NVP pouch cell are lower than 100%, which is caused by the NVP cathode with relatively low CEs of 98.8% and the un-optimized N/P ratio [61, 62]. Figure 3f demonstrates that the assembled full cell delivers a high energy density of 181.9 Wh kg^−1^ at a power density of 45.5 W kg^−1^, and even 37.9 Wh kg^−1^ at a power density of 1119.5 W kg^−1^ (based on the total mass of the cathode and anode). Even at − 60 °C, the full cell still delivers a capacity of 190.4 mAh g^−1^ at 1 A g^−1^ (Figs. 3g and S37). Totally, the achieved performances illustrate that the as-fabricated Bi/CNRs-15//NVP system possesses outstanding temperature adaptability, fast charging capability, and ultralong lifespan at ultralow temperature, providing evidences of Bi/CNRs-15 as a feasible anode for practical applications in low-temperature SIBs.

### Charge Storage Kinetics

**Fig. 4:**
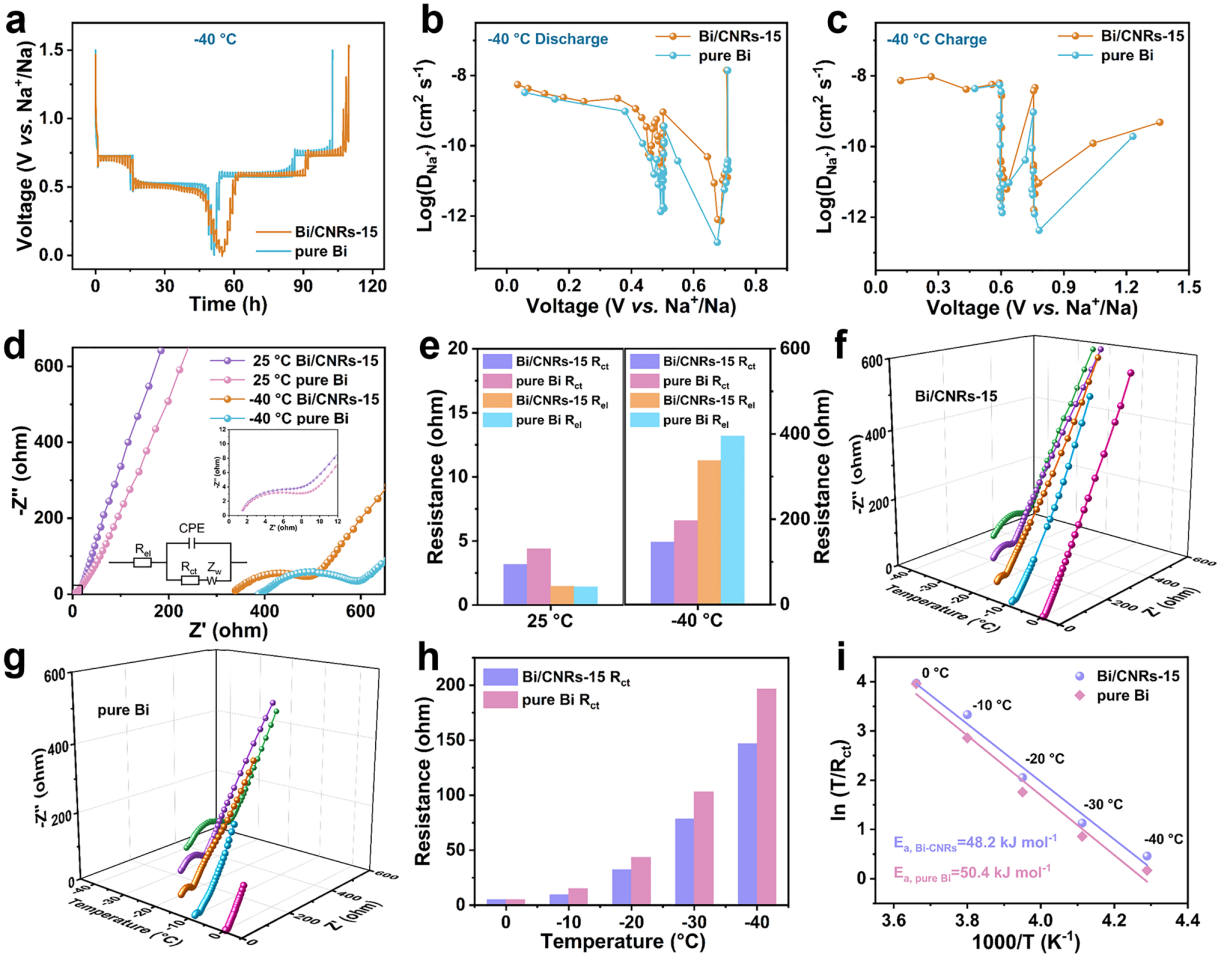
Electrochemical kinetics analysis at various temperatures. **a** GITT voltage profiles of the Bi/CNRs-15 and pure Bi electrodes at − 40 °C. Na^+^ diffusion coefficients of Bi/CNRs-15 and pure Bi during the **b** discharging and **c** charging processes at − 40 °C. **d** EIS plots of the fresh Bi/CNRs-15 and pure Bi electrodes at 25 and − 40 °C, where the inset shows corresponding equivalent circuit diagram and magnified view corresponding to the black box. **e** Comparisons of *R*_ct_ and *R*_el_ of the Bi/CNRs-15 and pure Bi electrodes at 25 (left) and − 40 °C (right). Temperature-dependent EIS study of** f** Bi/CNRs-15 and **g** pure Bi from − 40 to 0 °C. **h** Comparisons of *R*_ct_ of Bi/CNRs-15 and pure Bi at various temperatures. **i** Arrhenius curves and calculated *E*_a_ of Bi/CNRs-15 and pure Bi

The detailed Na^+^ diffusion kinetics of Bi/CNRs-15 and pure Bi were assessed by the galvanostatic intermittent titration technique (GITT) at both 25 and − 40 °C. Clearly, the slope region possesses higher Na^+^ diffusion coefficient (*D*) due to fast Na^+^ adsorption on the surface [63], while the plateau region exhibits lower *D* because of sluggish alloying/dealloying reactions. As the temperature drops to − 40 °C, the *D* values of Bi/CNRs-15 and pure Bi are in ranges of 1.4 × 10^–8^ to 7.4 × 10^–13^ and 1.4 × 10^–8^ to 3.7 × 10^–14^, respectively (Fig. 4a-c). In comparison with pure Bi, the enhanced *D* values of Bi/CNRs-15 under room temperature (Fig. S41) and low temperature, especially in the plateau region, are well consistent with unprecedented rate capability and also support the positive effect of nanoparticles driven by HTS on the kinetics of Na storage. Moreover, the Bi/CNRs-15 anode presents smaller Δ*Eτ* (instantaneous potential change during the constant current pulse) and Δ*E*s (steady-state potential change by the current pulse) than the pure Bi anode (Fig. S42), which indicates better Na^+^ diffusion ability of Bi/CNRs-15 than pure Bi.

Electrochemical impedance spectroscopy (EIS) measurements were conducted to quantitatively identify the charge transfer resistances and deeply comprehend the superior reaction kinetics of Bi/CNRs-15 [64]. One semicircle in high and middle frequencies and a straight line in low frequency can be observed in the Nyquist plots of Bi/CNRs-15 and pure Bi, implying similitude electrochemical processes in these two electrodes. The fitting results of charge transfer resistance (*R*_ct_) and electrolyte resistance (*R*_el_) values are quantified with the equivalent circuit. The *R*_ct_ value decreases gradually with increasing cycles and reaches an extremely low value of 1.2 Ω after 50 cycles (Fig. S43), suggesting that the Bi/CNR-15 electrode has excellent stability and maintains high electrochemical reaction kinetics. As presented in Fig. 4d, compared with room temperature, the steep rise of *R*_el_ at − 40 °C demonstrates low ionic conductivity caused by increased viscosity of the electrolyte [65]. We can also find that *R*_ct_ of Bi/CNRs-15 is slightly lower than that of pure Bi at 25 °C while much lower at − 40 °C, which indicates that Bi/CNRs-15 has a distinct advantage particularly under low-temperature conditions (Fig. 4e). The temperature-dependent EIS was further carried out to measure the values of *R*_ct_ from − 40 to 0 °C (Figs. 4f-h and S45). Based on the Arrhenius equation [65, 66],

1 $$T/R_{{{\text{ct}}}} = {\text{ A}}\,{\text{exp}}\left( { - E_{{\text{a}}} /{\text{R}}T} \right)$$

where *T* is the temperature, A is a constant and R is the gas constant. The activation energy (*E*_a_) for charge transfer (Fig. 4i) of Bi/CNRs-15 is calculated to be 48.2 kJ mol^−1^, which is lower than that of pure Bi (50.4 kJ mol^−1^). The above results verify that nanomanufacturing and hierarchical porous structure are more conducive to reducing the charge transfer barrier and achieving fast reaction kinetics, which are vital to acquire incredible fast charging capability at extremely low temperature.

### Stable SEI Component

**Fig. 5:**
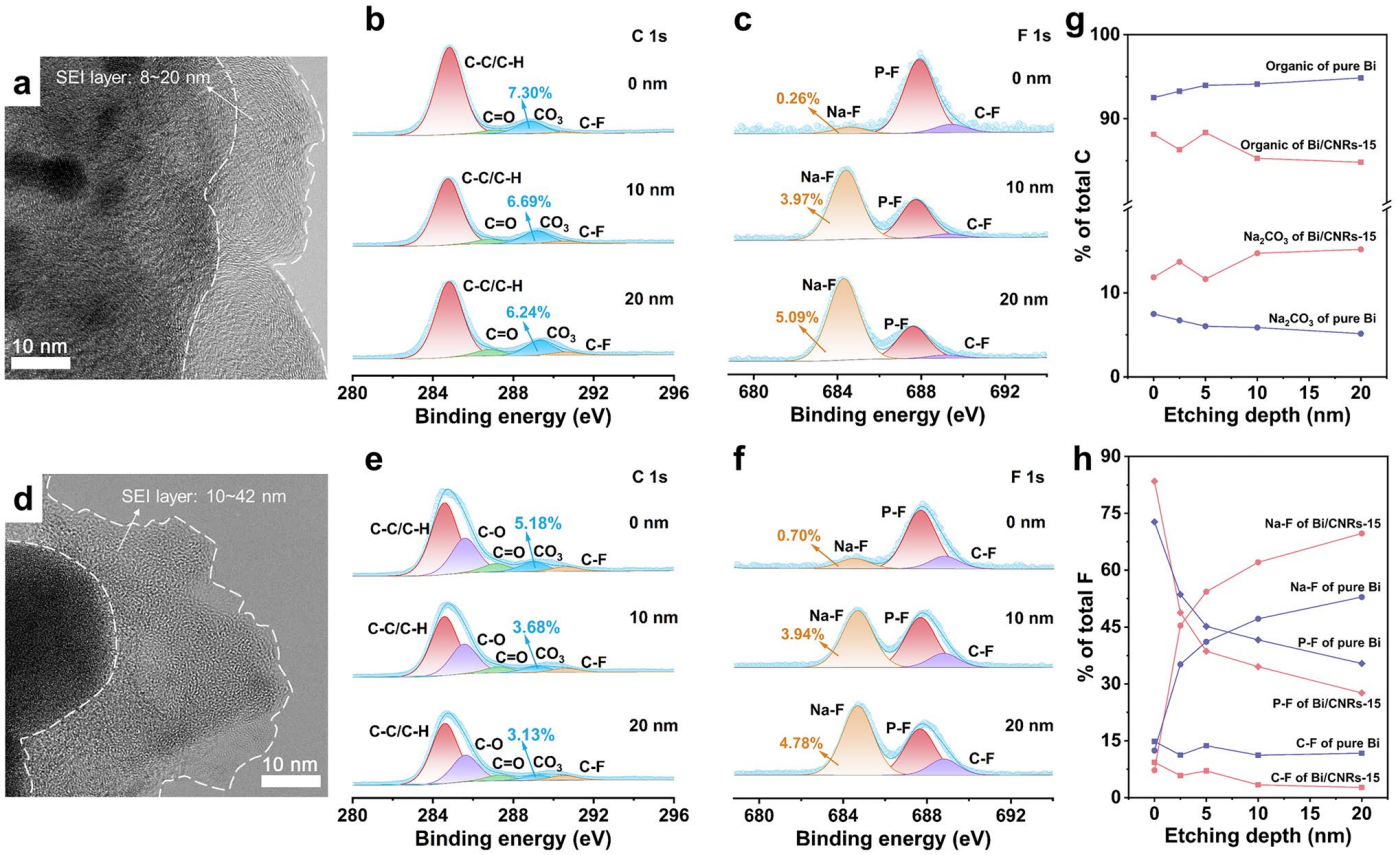
Surface composition analysis of the Bi/CNRs-15 and pure Bi electrodes operating at − 40 °C. **a** HRTEM image of the Bi/CNRs-15 electrode after 5 cycles at 0.1 A g^−1^. Depth-profiling XPS spectra of **b** C 1*s* and** c** F 1*s* on the Bi/CNRs-15 electrode. **d** HRTEM image of the pure Bi electrode after 5 cycles at 0.1 A g^−1^. Depth-profiling XPS spectra of** e** C 1*s* and** f** F 1*s* on the pure Bi electrode. The proportion of SEI components calculated from **g** C 1*s* and **h** F 1*s* spectra

As an inert layer formed from chemical and electrochemical reactions of components in the electrolyte, SEI plays a critical role in determining the performance of batteries [67]. Meanwhile, a cold condition can convert the reaction of electrolyte decomposition and results in the formation of metastable SEI with dominant organics, which greatly decelerates the migration of Na^+^ [68]. In order to further study the relationship between SEI and Na storage behavior, TEM and XPS measurements with different etching depths were carried out for both Bi/CNRs-15 and pure Bi electrodes after 5 cycles at − 40 °C. As shown in Fig. 5a, d, the SEI layer of Bi/CNRs-15 is thin (the thickness of 8–20 nm) and homogeneous, whereas that of pure Bi is thicker (the thickness of 10–42 nm) and uneven. By deeply analyzing the high-resolution spectra of C 1*s*, F 1*s*, and O 1*s* (Figs. 5b, c, e, f and S46), the C 1*s* peaks at 284.8 eV (C–C/C–H), 285.7 eV (C–O), 287.1 eV (C=O), and O 1*s* peaks located at 533.2 eV (C=O) are associated with organic compounds primarily derived from the decomposition of solvents [69, 70]. The peaks at 289.1 eV (Na_2_CO_3_) of C 1*s* spectra, the peaks located at 529.7 eV (Na_2_O) of O 1*s* spectra and the peaks at 684.1 eV (Na-F) are assigned to inorganic components produced from Na^+^-solvent-PF_6_^−^ reduction products and the decomposition of NaPF_6_ [71, 72]. The peaks at 290.6 eV (C-F) of C 1*s* spectra and the peaks located at 689.1 eV (C-F) of F 1*s* spectra illustrate that fluorinated organic compounds reside in SEI layer [73]. A small amount of robust fluorinated organic compounds are highly efficacious to preclude the continuous side reactions of SEI components [74]. As observed in Fig. 5g, h, the concentration of organic species in SEI of pure Bi is commonly higher than that of Bi/CNRs-15, demonstrating more decompositions of solvent existing in the pure Bi electrode. To the extent that the total peak area ratios of Na_2_CO_3_, Na_2_O, and Na-F peaks stand for inorganic species in SEI layer. With increasing etching depths, the proportions of inorganic compounds in SEI on the Bi/CNRs-15 electrode gradually step up and are higher than those in SEI of pure Bi throughout. Specifically, NaF with high lowest unoccupied molecular orbital level and decent band gap is a good electron insulator to block electrons [75]. Na_2_CO_3_ with relatively high ionic conductivity is conducive to the transport of Na^+^ [76]. These two inorganic components can synergize with each other to construct a robust SEI of the anode side. Furthermore, as the sputtering depth increases, the soaring rise in proportion of NaF indicates that the inner SEI layer is mainly composed of inorganic species. The continuous and compact inner SEI layer can effectively buffer the volume expansion and improve the cycling lifetime at low temperature [68]. Overall, more inorganic component in SEI layer on the Bi/CNRs-15 electrode greatly interprets the reason for better rate performance and cycling stability of Bi/CNRs-15 than pure Bi.

### High-Rate Activation

**Fig. 6:**
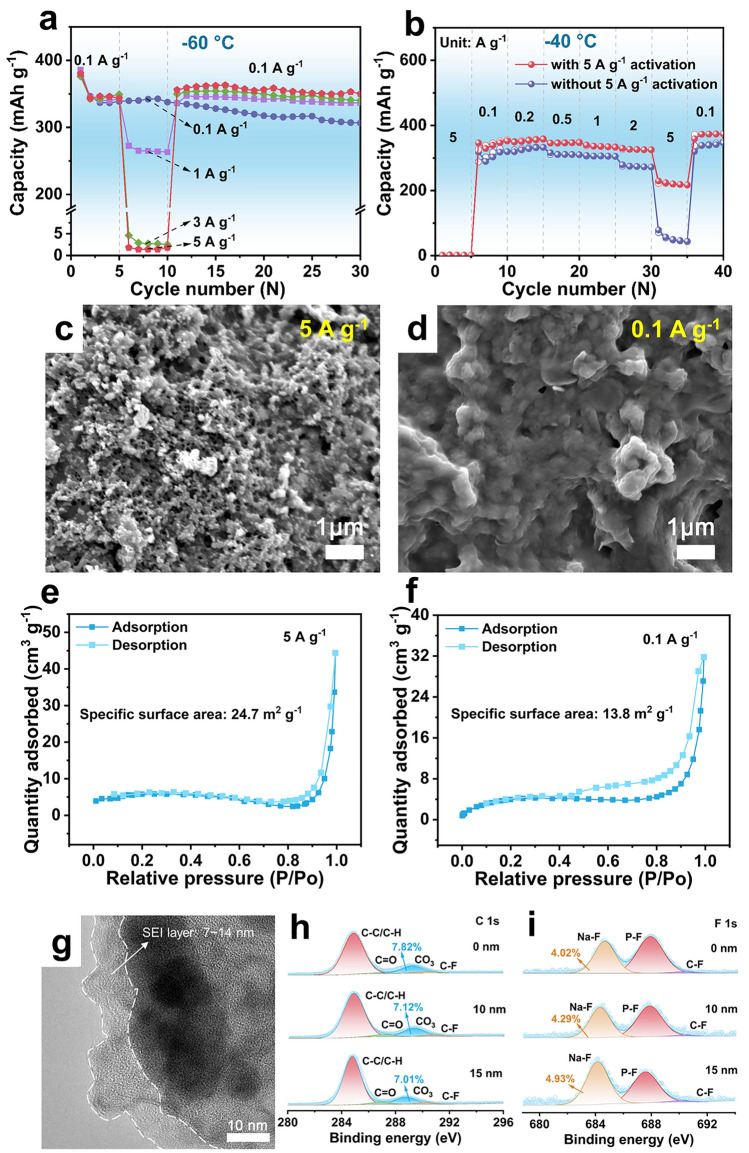
High-rate activation. **a** Capacity comparisons after high-rate activation (cycled at 0.1 A g^−1^ for 5 times, then 0.1/1/3/5 A g^−1^ for 5 cycles, finally returned to cycling at 0.1 A g^−1^). **b** Rate performance of Bi/CNRs-15 with a high mass loading (about 1 mg cm^−2^). **c** SEM image of the Bi/CNRs-15 electrode after 5 cycles at 5 A g^−1^. **d** SEM image of the Bi/CNRs-15 electrode after 5 cycles at 0.1 A g^−1^. **e** N_2_ adsorption/desorption isotherms of the Bi/CNRs-15 electrode after 5 cycles at 5 A g^−1^. **f** N_2_ adsorption/desorption isotherms of the Bi/CNRs-15 electrode after 5 cycles at 0.1 A g^−1^. **g** HRTEM image of the Bi/CNRs-15 electrode after 5 cycles at 5 A g^−1^. Depth-profiling XPS spectra of **h** C 1*s* and **i** F 1*s* on the Bi/CNRs-15 electrode after 5 cycles at 5 A g^−1^

Shockingly, we found that the capacity can be restored and even higher at 0.1 A g^−1^ after losing almost all capacities at 5 A g^−1^ (Fig. 2e). To further explore such an unusual phenomenon named “negative fading”, we tested the cycling performance at 0.1 A g^−1^ after high-rate activation (Fig. 6a). Compared with the performance without high-rate activation, Bi/CNRs-15 after high-rate (1/3/5 A g^−1^) cycling shows capacity increment and stable capacity retention to some extent. The Bi/CNRs-15 electrode with a high mass loading (Fig. 6b) also represents better rate property after high-rate activation at − 40 °C. As shown in Figs. 6c, d, and S47, the electrode activated after high rate of 5 A g^−1^ evolved into smaller-sized coral-like porous nanostructures due to the high mechanical stress during repeated volume changes. To further study the specific surface area of the Bi/CNRs-15 electrode after activation, nitrogen adsorption–desorption test was implemented. It is found that the specific surface area of the Bi/CNRs-15 composite is relatively small (6.8 m^2^ g^−1^, Fig. S48) before activation. However, after 5 cycles at 5 A g^−1^, the specific surface area of Bi/CNRs-15 composite increases significantly to 24.7 m^2^ g^−1^ (Fig. 6e), which is larger than that after 5 cycles at 0.1 A g^−1^ (13.8 m^2^ g^−1^, Fig. 6f). The expanded specific surface area induced by morphology evolution creates additional active sites for interfacial reaction to improve the kinetic properties, resulting in the capacity increment [77, 78]. Ulteriorly, the instantaneous surface activation under large current can dramatically reduce the side reaction between the anode and the electrolyte [79]. The EIS tests were also conducted to further understand the nature behind the excellent electrochemical performance of high-rate activation. As shown in Fig. S49, the value of *R*_ct_ after 5 cycles at 5 A g^−1^ is only 74.5 Ω, much lower than that after 5 cycles at 0.1 A g^−1^ (174.6 Ω). Figure 6g-i demonstrates that the SEI layer of the Bi/CNRs-15 electrode after 5 cycles at 5 A g^−1^ (the thickness of 7–14 nm) is thinner than that of the Bi/CNRs-15 electrode at 0.1 A g^−1^ (the thickness of 8–20 nm). The dense inorganic species dominate in the inner layer, while a small number of organic compounds are distributed near the surface, showing a layered structure. This is beneficial for Na^+^ transfer and enhances the rate capability of the Bi/CNRs-15 electrode after high-rate activation [80, 81]. Overall, due to thinner and more homogeneous SEI layer after high-rate activation and more active sites induced by morphology evolution for redox reaction, the Bi/CNRs-15 electrode after high-rate activation delivers better sodium storage performance. This unique perspective can be used for obtaining high capacity and superior stability of the Bi-based electrodes at low temperature by creating new electrochemically active sites under large current.

## Conclusions

In this work, a Bi/CNRs-15 hybrid has been elaborately constructed through ultrafast HTS method. The unique HTS process with rapid elevating/quenching rates and short heating time in seconds enables the synthesis of well-dispersed and high-loading ultrafine metallic nanoparticles. As revealed, Bi/CNRs-15 not only exhibits high Na^+^ diffusion coefficient, but also possesses low charge transfer activation energy. Being used as the anode material for SIBs, Bi/CNRs-15 displays unprecedented fast-charging ability at ultralow temperature. The full cell (Bi/CNRs-15//NVP) also delivers a high energy density and a promising power density at − 40 °C. Furthermore, a stable and homogenous SEI layer with more inorganic species could improve the structural stability and boost rate kinetics in some degree, which is unraveled by X-ray photoelectron spectroscopy etching. More importantly, an abnormal phenomenon at low temperature of capacity increment named “negative fading” is explored. The guidance of this work makes it possible for further applications of low-temperature fast-charging SIBs.

## Supplementary Information

Below is the link to the electronic supplementary material.

Supplementary file1 (DOCX 13660 kb)
