# Supplementary material for: Ideal Bi-Based Hybrid Anode Material for Ultrafast Charging of Sodium-Ion Batteries at Extremely Low Temperatures
Source: Nanomicro Lett. 2024 Nov 13;17:60. doi: 10.1007/s40820-024-01560-9 (PMC11557858; doi:10.1007/s40820-024-01560-9)
Supplement: Supplementary file 1 — Supplementary file1 (DOCX 13660 kb) [file 40820_2024_1560_MOESM1_ESM.docx]

Supporting Information for

**Ideal Bi-Based Hybrid Anode Material for Ultrafast Charging of Sodium-Ion Batteries at Extremely Low Temperatures**

Jie Bai^1^, Jian Hui Jia^1^, Yu Wang^1^, Chun Cheng Yang^1,^*, and Qing Jiang^1,^*

^1^Key Laboratory of Automobile Materials (Jilin University), Ministry of Education, School of Materials Science and Engineering, Jilin University, Changchun 130022, P. R. China

*Corresponding authors. E-mail: [ccyang@jlu.edu.cn](mailto:ccyang@jlu.edu.cn) (Chun Cheng Yang); [jiangq@jlu.edu.cn](mailto:jiangq@jlu.edu.cn) (Qing Jiang)

**Supplementary** **Figures**

**
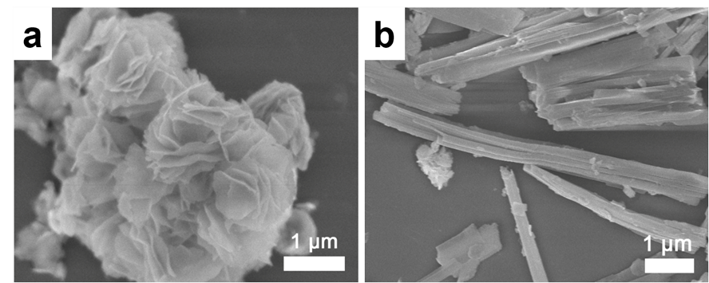
**

**Fig.** **S1** SEM images of **a** BiOI NSs and **b** Bi-MOFs

**
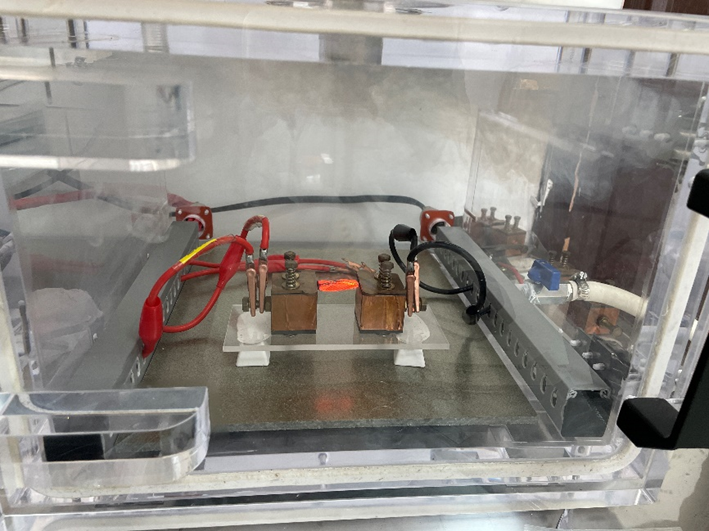
**

**Fig. S2** Photograph of the HTS device

**
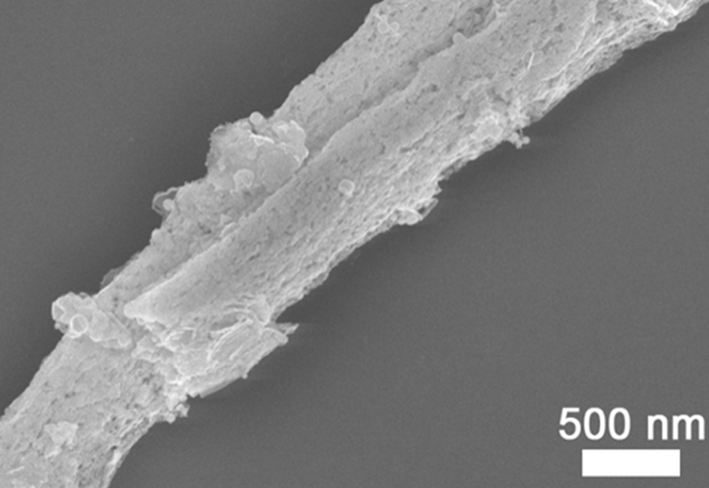
**

**Fig. S3** SEM image of Bi/CNRs-15

**
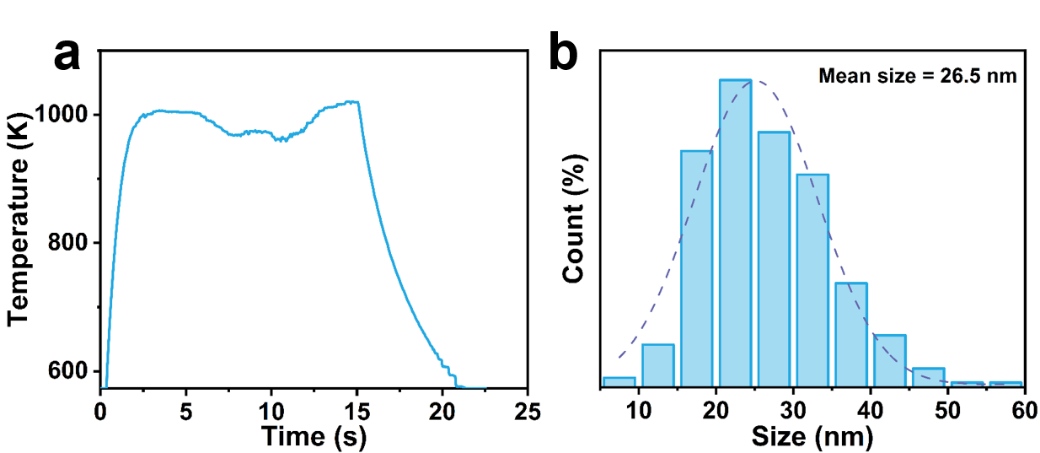
**

**Fig. S4** **a** Transient temperature profile and **b** nanoparticle size distribution of Bi/CNRs-15

**
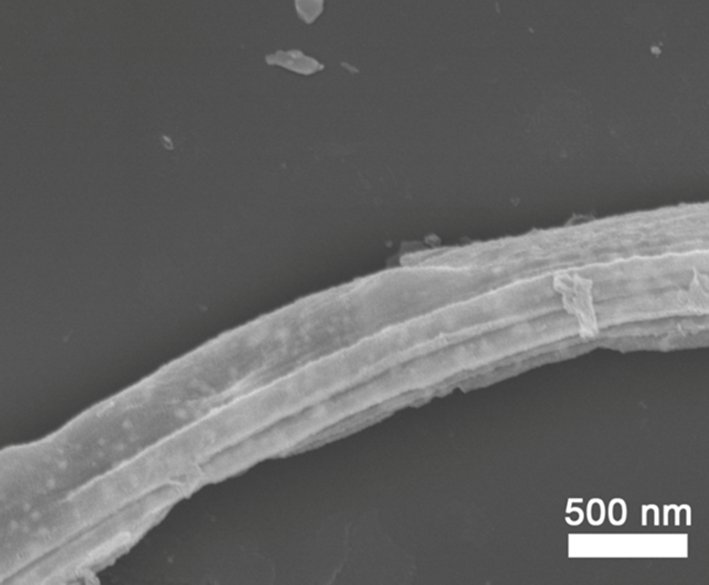
**

**Fig. S5** SEM image of Bi/CNRs-TFA

**
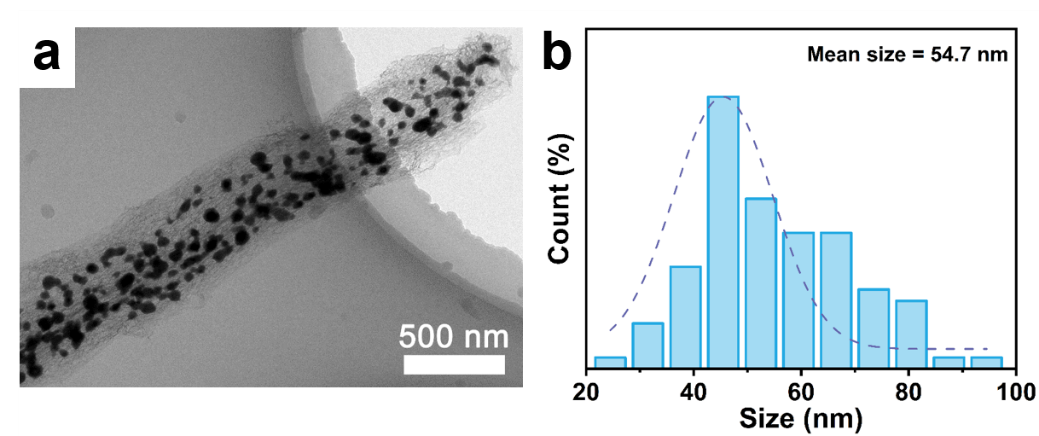
**

**Fig. S6 a** TEM image and **b** nanoparticle size distribution of Bi/CNRs-TFA

**
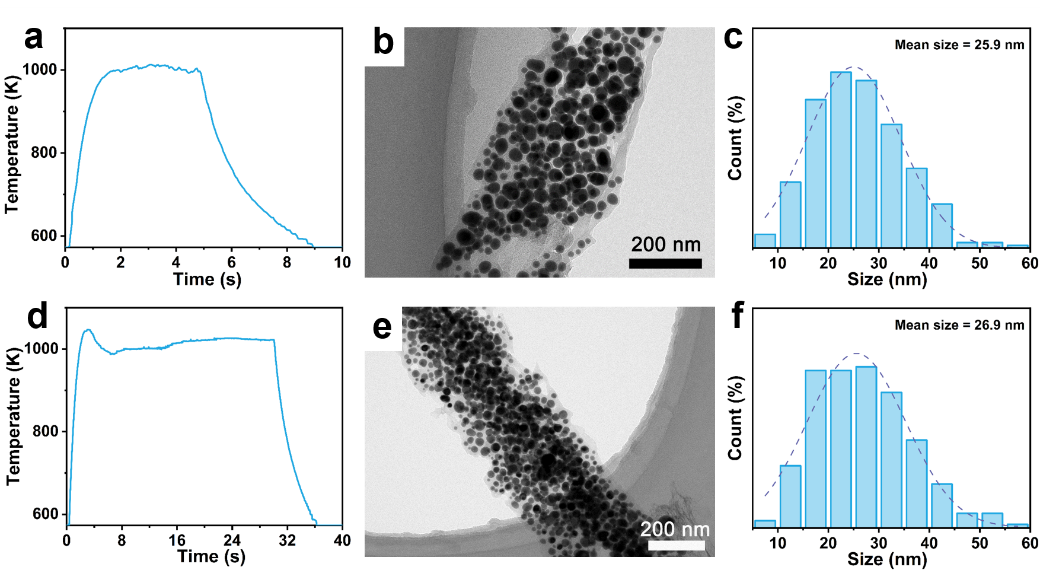
**

**Fig. S7** **a** The transient temperature profile, **b** TEM image and **c** size distribution of Bi/CNRs-5. **d** The transient temperature profile, **e** TEM image and **f** size distribution of Bi/CNRs-30

**
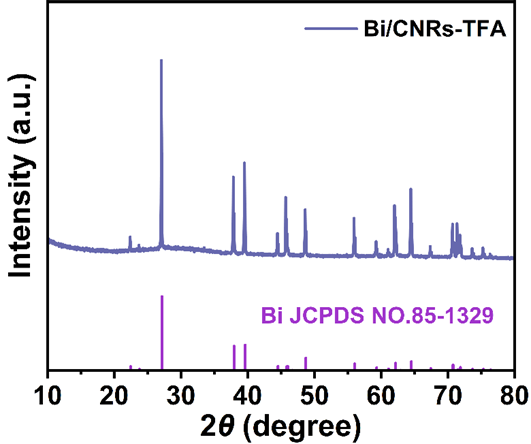
**

**Fig. S8** XRD pattern of Bi/CNRs-TFA

**
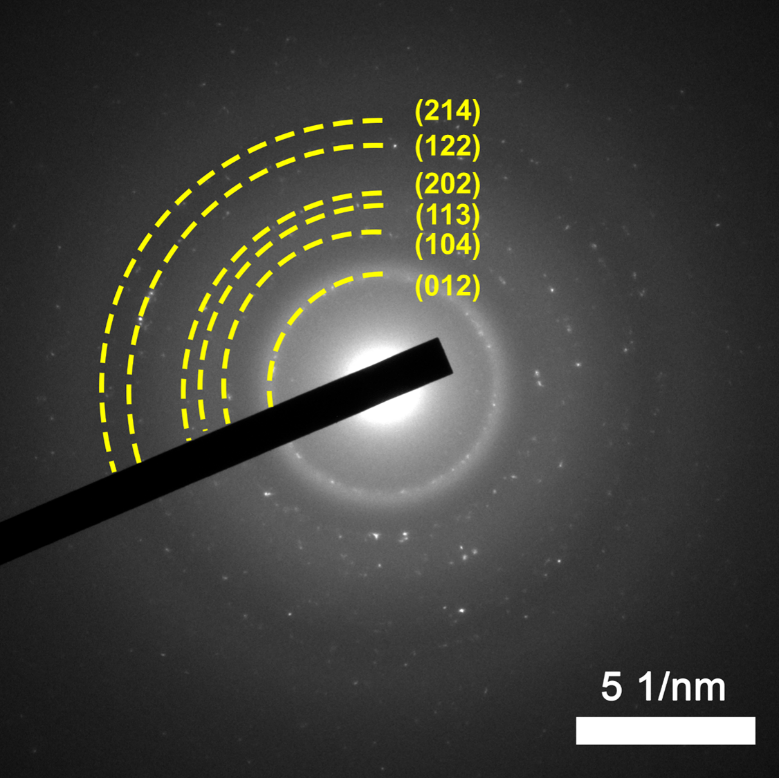
**

**Fig. S9** SAED pattern of Bi/CNRs-15

**
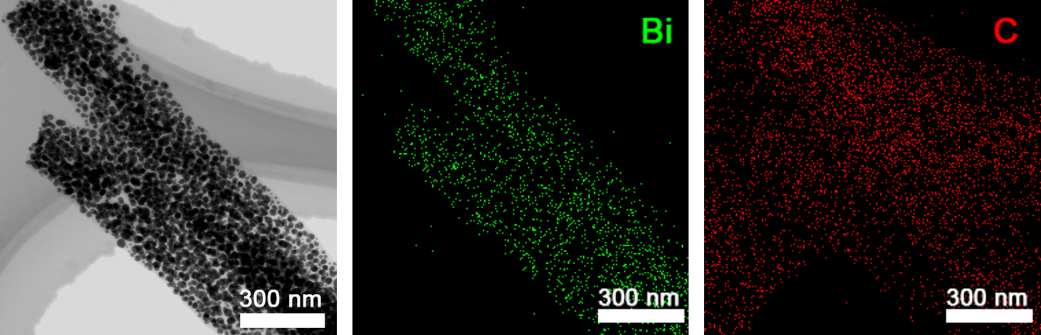
**

**Fig. S10** BF-STEM image of Bi/CNRs-15 and corresponding elemental mappings for Bi and C elements

**
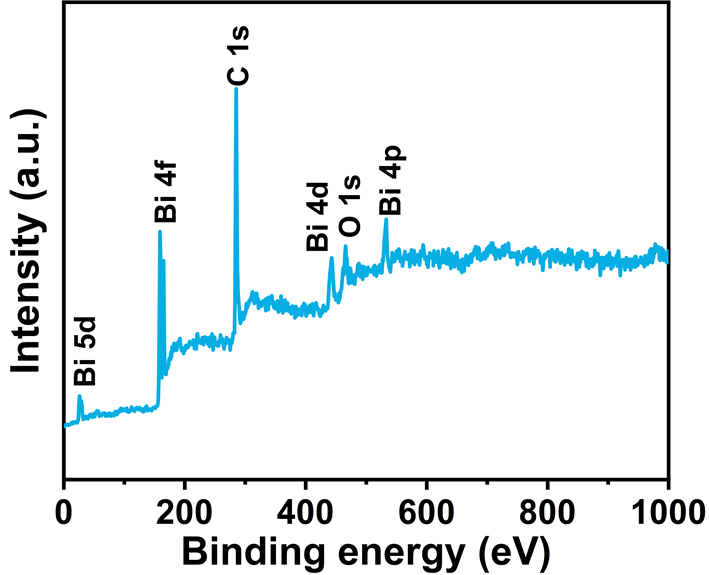
**

**Fig. S11** XPS survey spectrum of Bi/CNRs-15

**
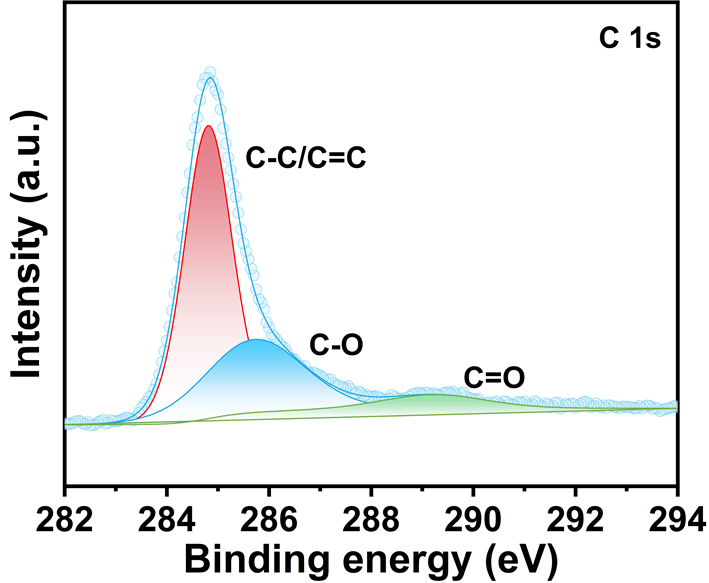
**

**Fig. S12** C 1s spectrum of Bi/CNRs-15


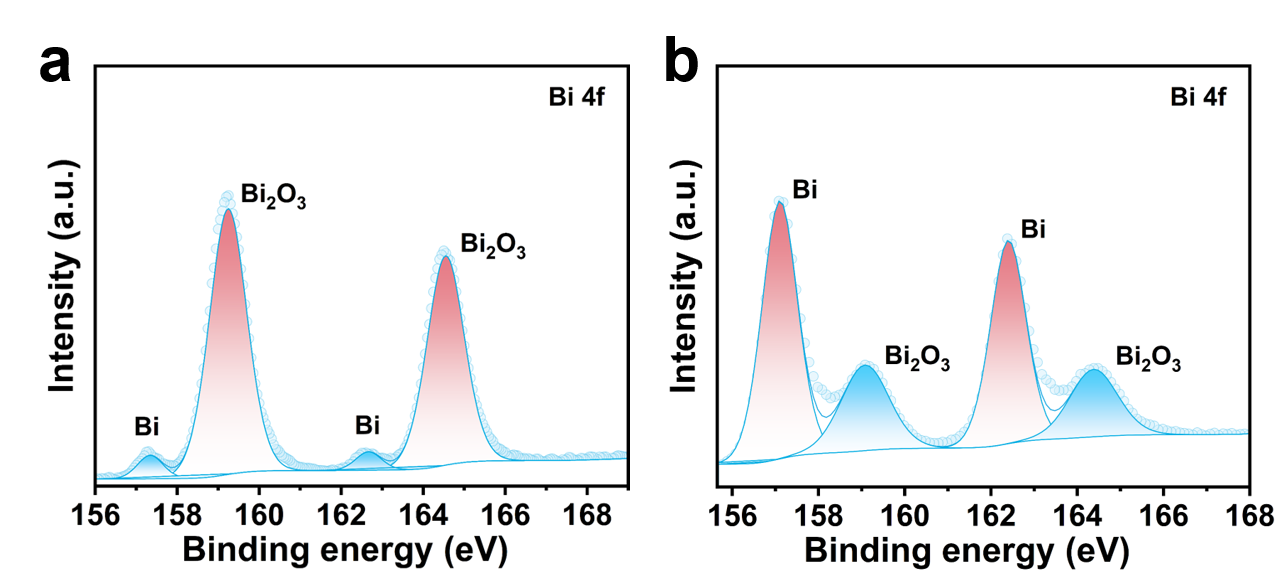


**Fig. S13 a** Bi 4f spectrum of Bi/CNRs-15. **b** Bi 4f spectrum of Bi/CNRs-15 with the etching depths of 10 nm

**
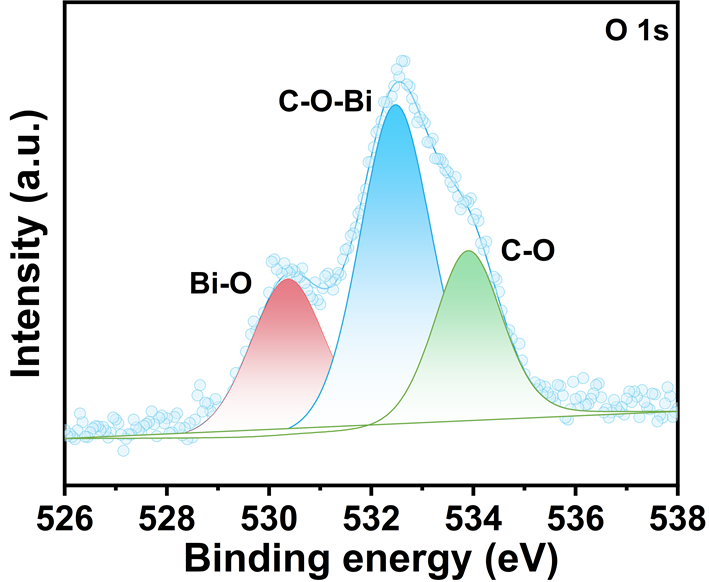
**

**Fig. S14** O 1s spectrum of Bi/CNRs-15

**
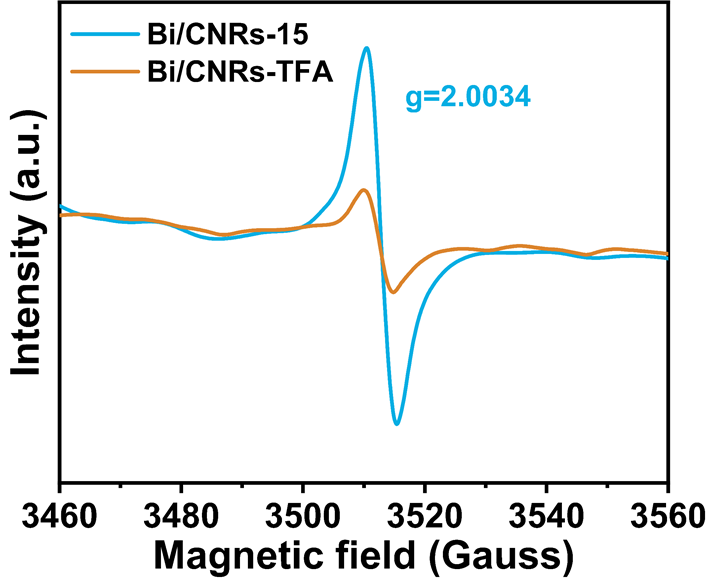
**

**Fig. S15** EPR spectra of Bi/CNRs-15 and Bi/CNRs-TFA

**
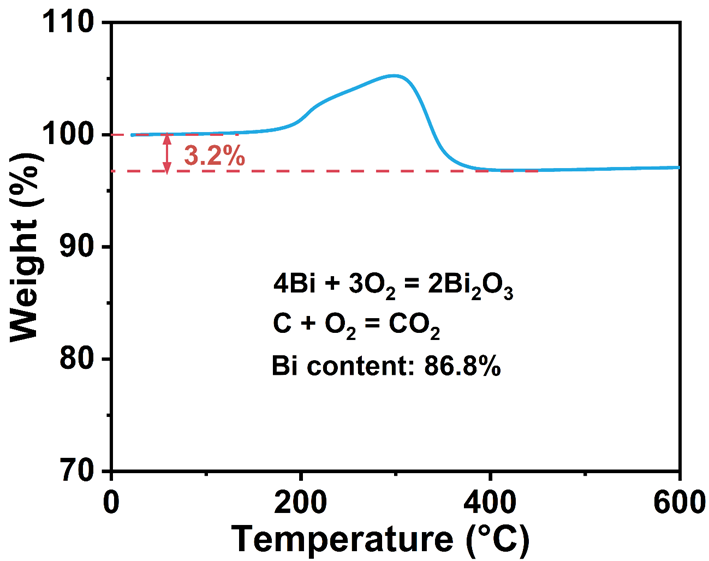
**

**Fig. S16** TGA curve of Bi/CNRs-15

The reason for the weight percentage increase in the temperature range of 200 to 300 °C is that the rate of Bi oxidation is faster than the rate of evaporation of adsorbed water and loss of C to CO_2_ gas [Ref. 50 of the text]. The content of metallic Bi in the Bi/CNRs-15 composite is calculated to be 86.8 wt% from the equation below.

Bi (wt%) =$\frac{\text{2*molecular weight of Bi}}{\text{molecular weight of }\text{Bi}_{\text{2}}\text{O}_{\text{3}}}\text{ }\text{×}\text{ }\frac{\text{weight of }\text{Bi}_{\text{2}}\text{O}_{\text{3}}}{\text{weight of Bi/CNRs-15}}\text{ }\text{×}\text{ 100\% = }\frac{\text{2*208.98}}{\text{465.96}}\text{ }\text{×}\text{ }\frac{\text{1-0.032}}{\text{1}}\text{ }\text{×}\text{ 100\% = 86.8\%}$

**
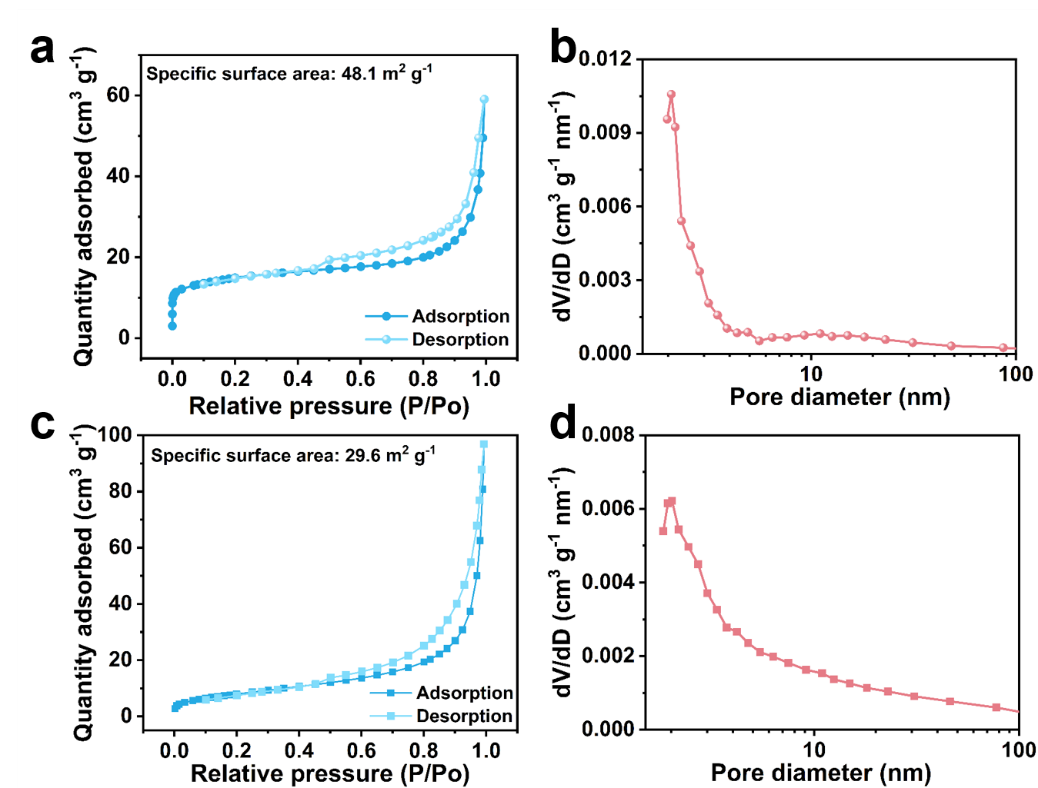
**

**Fig. S17 a** N_2_ adsorption/desorption isotherms and **b** pore size distribution of Bi/CNRs-15. **c** N_2_ adsorption/desorption isotherms and **d** pore size distribution of Bi-MOFs

**
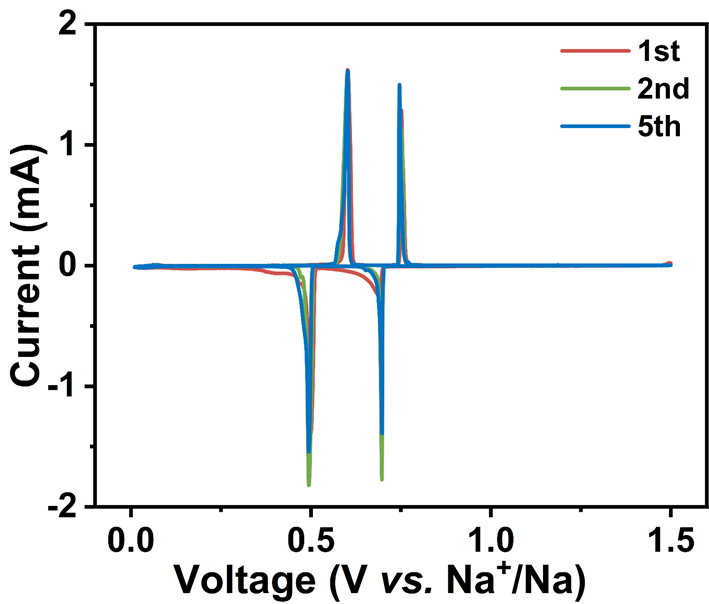
**

**Fig. S18** CV curves of Bi/CNRs-15 at 0.1 mV s^-1^

Two cathodic peaks at 0.49 and 0.70 V are related to the stepwise alloy reactions from Bi to NaBi and further to Na_3_Bi. The sharp redox peaks and highly overlapped CV curves indicate fast kinetics and superior reversibility of Bi/CNRs-15.

**
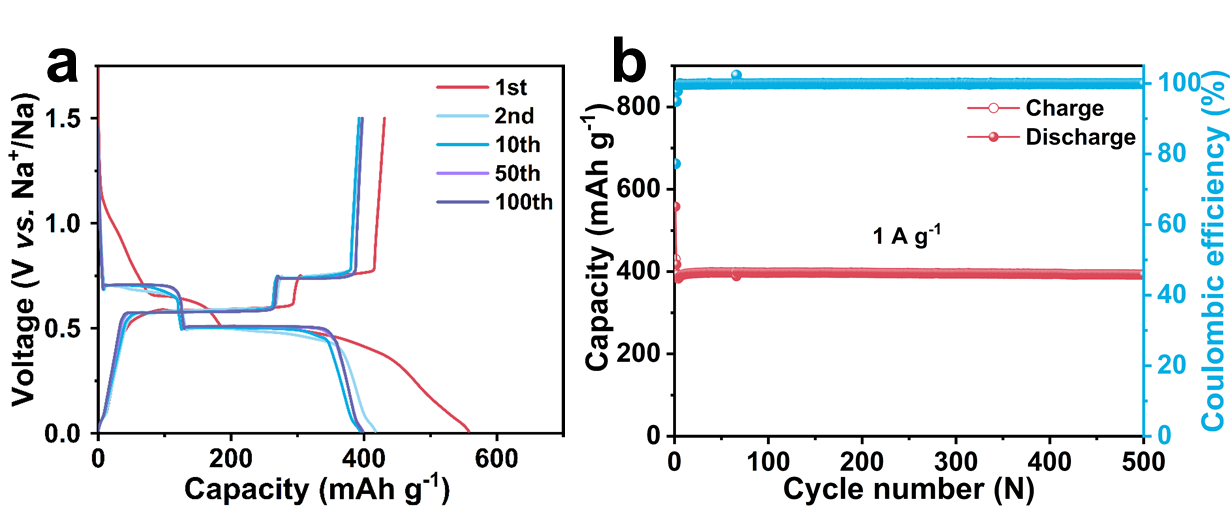
**

**Fig. S19 a** Galvanostatic charge/discharge curves and **b** cycling performance of Bi/CNRs-15 at 1 A g^-1^

The Bi/CNRs-15 electrode achieves the initial discharge/charge capacities of 557.7 and 430.8 mAh g^-1^, respectively, corresponding to a high initial Coulombic efficiency (ICE) of 77.2%. The irreversible capacity in the 1^st^ cycle is inferred to be the formation of the solid electrolyte interphase (SEI) film [S1]. The ICE values can potentially be improved according to the following strategies, such as electrolyte engineering (optimizing the formula of electrolyte and adding the electrolyte additives), binder optimization (developing multifunctional binders) and pre-sodiation (mixing the anode materials with sodium metal) [S2, S3]. Meanwhile, the discharge curves display pronounced plateaus at 0.70 and 0.50 V while plateaus at 0.60 and 0.75 V in the charge curves, which are completely in conformity with the CV curves. A reversible capacity of 393 mAh g^-1^ can be achieved after 500 cycles with a high capacity retention of 94.2% relative to the second cycle.

**
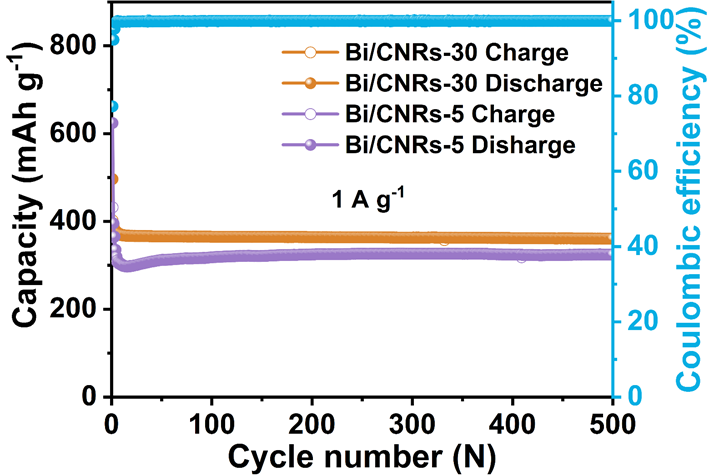
**

**Fig. S20** Cycling performances of Bi/CNRs-5 and Bi/CNRs-30 at 1 A g^-1^

The Bi/CNRs-5 and Bi/CNRs-30 electrodes provide lower capacities of 324.5 mAh g^-1^/81.9% and 360.7 mAh g^-1^/92.1%, respectively, compared with Bi/CNRs-15.

**
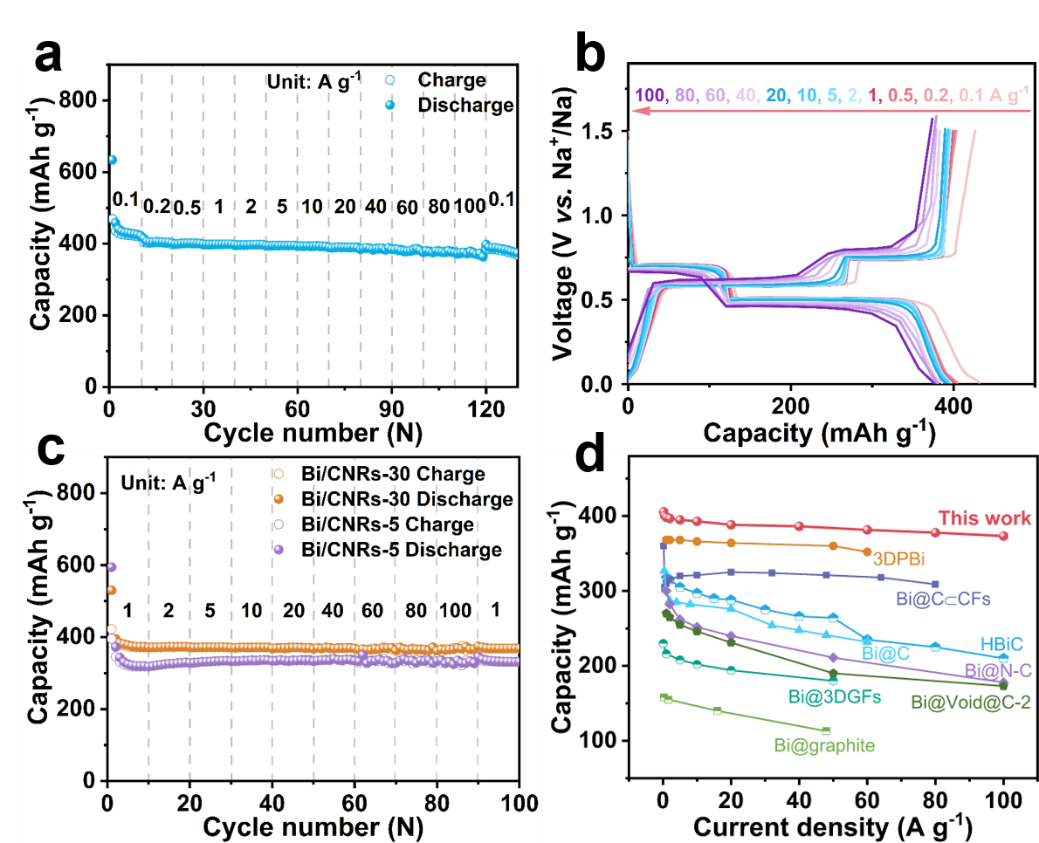
**

**Fig. S21** **a** Rate performance and **b** charge/discharge curves at various rates of Bi/CNRs-15. **c** Rate performances of Bi/CNRs-5 and Bi/CNRs-30. **d** Comparisons of rate performance of Bi/CNRs-15 with reported Bi-based SIB anodes

Moreover, Bi/CNRs-15 also exhibits the best rate performance compared with Bi/CNRs-5 and Bi/CNRs-30. At relatively low current densities (0.2, 0.5, and 1 A g^-1^), the average rate capacity reaches 405.6, 400.7 and 398.3 mAh g^-1^, respectively. Even with a sharp increase in the current to 100 A g^-1^ (only 13 s to complete full charge or discharge), the capacity of Bi/CNRs-15 displays tiny fluctuation and still remains stable at 373.4 mAh g^-1^. Furthermore, two notable voltage plateaus still exist in the galvanostatic charge/discharge curves of Bi/CNRs-15 at 100 A g^-1^, suggesting low polarization. It is worth noting that the Bi/CNRs-15 electrode provides ultrahigh stability even at extremely large current, showing competitive rate property compared with reported Bi-based anodes for SIBs [S1-S8].

**
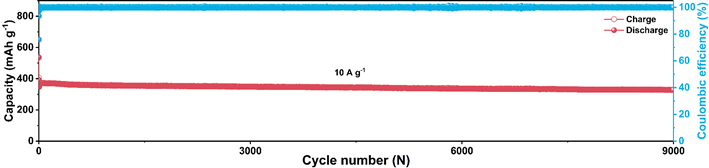
**

**Fig. S22** Long-term cycling performance of Bi/CNRs-15 at 10 A g^-1^

In order to further validate the sustainability of Bi/CNRs-15, the long-term cycling test at a high rate of 10 A g^-1^ was implemented, which remains a reversible capacity of 328.4 mAh g^-1^ after 9000 cycles with a decay rate of only 0.002% per cycle, illustrating the fast reaction kinetics and appealing cycling stability.

**
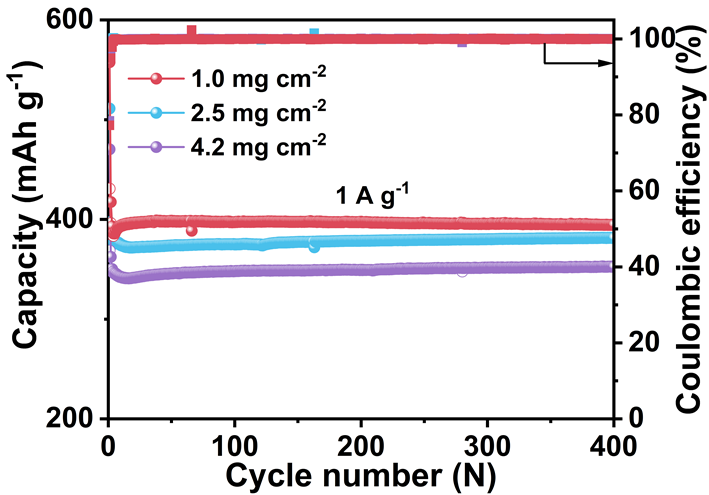
**

**Fig. S23** Cycling performance of the Bi/CNRs-15 electrode under different mass loadings (1.0, 2.5 and 4.2 mg cm^-2^) at 1 A g^-1^

Even under high mass loadings of 2.5 and 4.2 mg cm^-2^, the Bi/CNRs-15 electrode still has high discharge capacities of 381.5 and 352.5 mAh g^-1^ after 400 cycles under 1 A g^-1^, respectively. Compared with the mass loading of 1.0 mg cm^-2^ (394.6 mAh g^-1^ after 400 cycles under 1 A g^-1^), the capacities for 2.5 and 4.2 mg cm^-2^ only decay 13.1 and 42.1 mAh g^-1^, respectively. This indicates that the Bi/CNRs-15 electrode exhibits superior electrochemical performance even under high mass loadings.

**
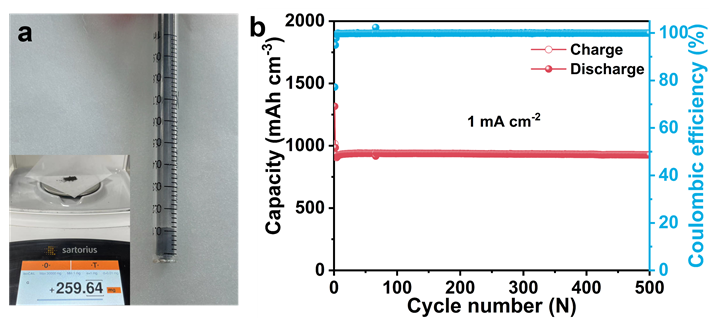
**

**Fig. S24 a** Volume of the Bi/CNRs-15 powder after 500 times vibration, and the inset shows related mass. **b** Cycling performance of Bi/CNRs-15 based on volumetric capacity at 1 mA cm^-2^.

The average tap density of Bi/CNRs-15 is calculated to be 2.36 g cm^-3^, and the volumetric capacity of the Bi/CNRs-15 electrode remains as high as 927.5 mAh cm^-3^ at 1 mA cm^-2^ after 500 cycles.

**
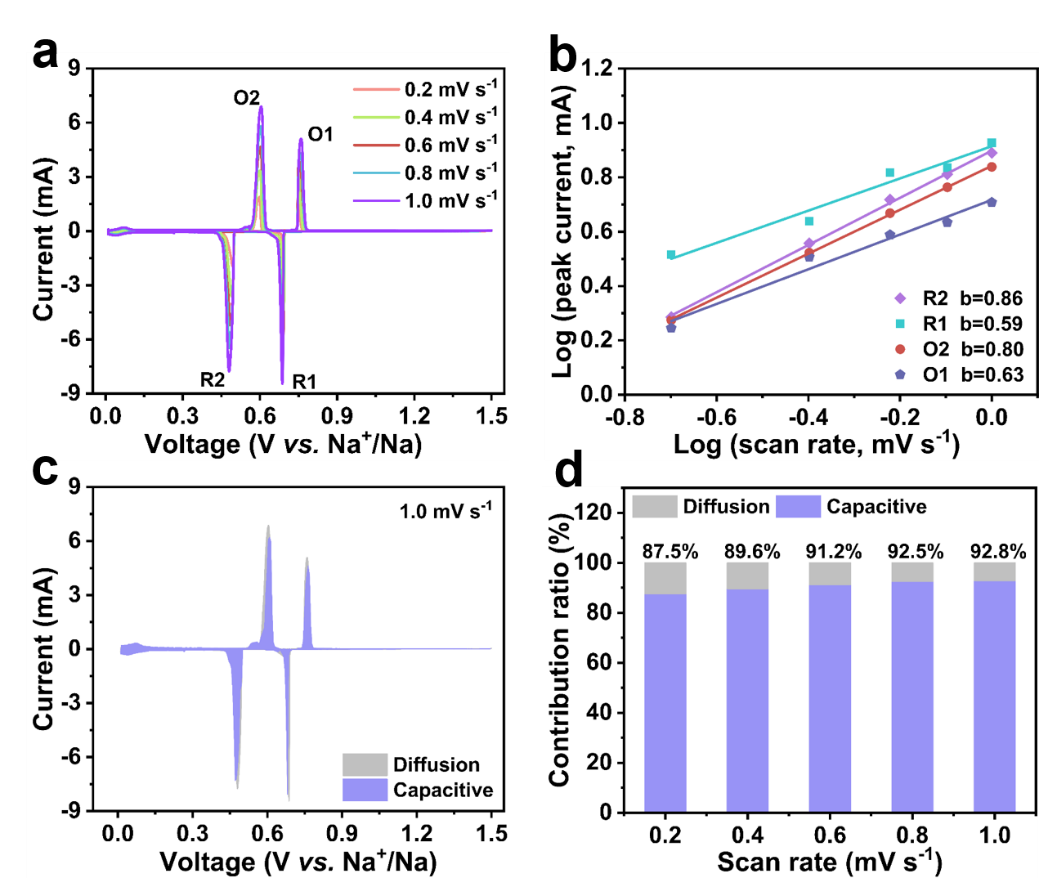
**

**Fig. S25 a** CV curves of Bi/CNRs-15 at various scan rates. **b** The determination of *b* value. **c** Contributions of the pseudocapacitance and diffusion at a scan rate of 1.0 mV s^-1^. **d** Contribution ratios of capacitive- and diffusion-controlled capacities at different scan rates

The redox peaks hold similar shapes and peak potentials at increased scan rates, suggesting a small polarization voltage and fast reaction kinetics. The relationship of the scanning rate (*v*) and peak current (*i*) can be described as [S9]:

*i* = *av^b^* (S1)

where both *a* and *b* are variables. Generally, the *b* values of 0.5 and 1.0 represent the completely diffusion-controlled process (Faradaic) and capacitive-dominated behavior, respectively. The quantitative contributions of capacitive (*k*_1_*v*) and diffusion process (*k*_2_*v*^1/2^) in Bi/CNRs-15 can be calculated based on the following equation [S9]:

*i*(V) *= k*_1_*v* + *k*_2_*v*^1/2^ (S2)

The sweep-rate-dependent CV technique was employed to explore remarkable high-rate performance at room temperature. Through fitting two couples of redox peaks, the *b* values are 0.86, 0.59, 0.80 and 0.63 for R1, R2, O2 and O1, respectively, which demonstrates that the redox process is joint controlled by the pseudocapacitance and diffusion [8]. With increasing the scan rate, the proportion of the capacitive contribution gradually increases from 87.5% to 92.8%. This reveals that carbon vacancies induced by ultrafast HTS can facilitate Na^+^ adsorption to endow extra capacities and fast kinetics [S5, S10].

**
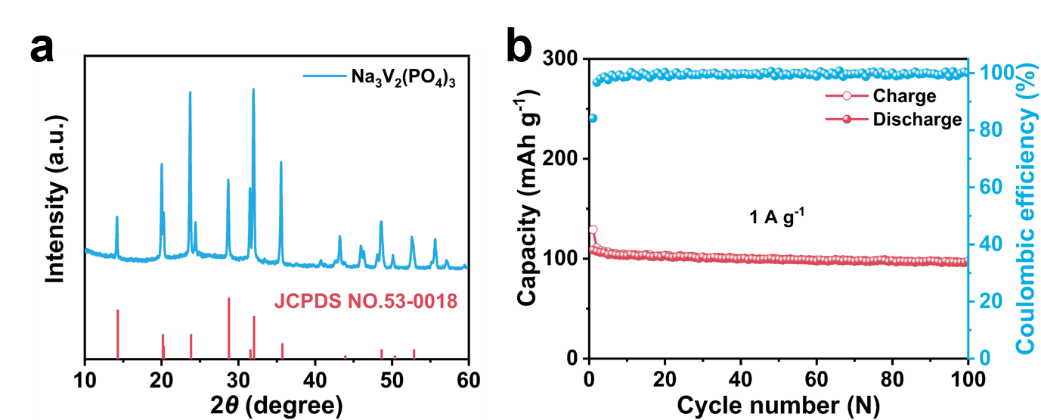
**

**Fig. S26 a** XRD pattern and **b** cycling performance of Na_3_V_2_(PO_4_)_3_ (NVP) at 1 A g^-1^

**
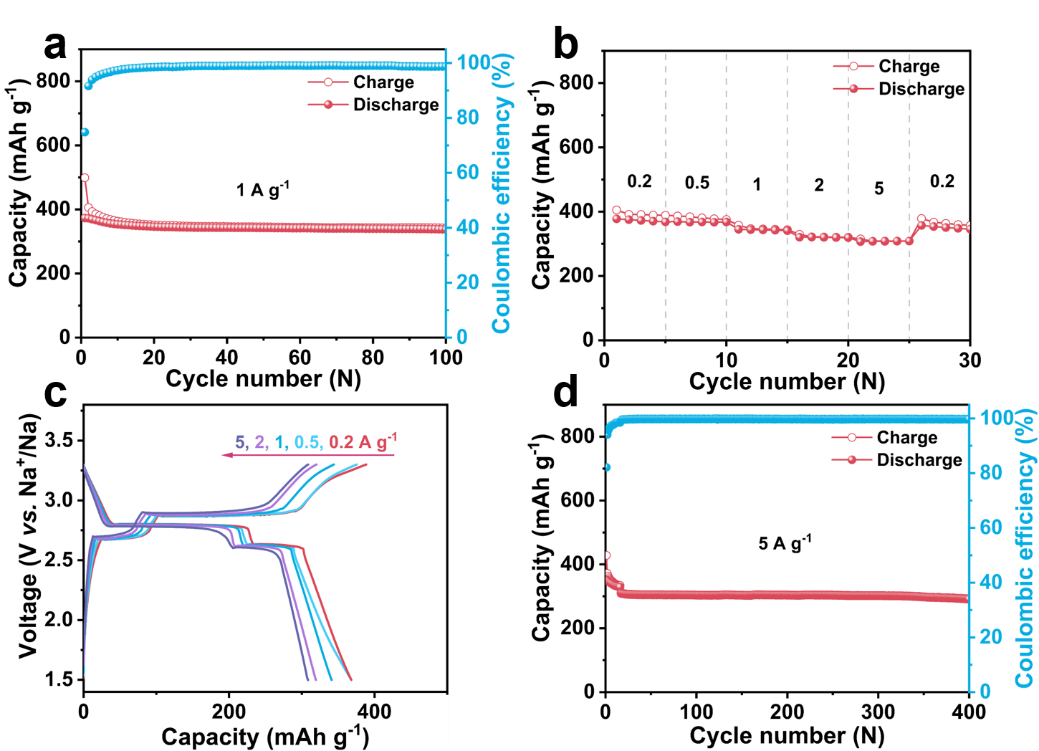
**

**Fig. S27** Electrochemical properties of the Bi/CNRs-15//NVP full cell at room temperature. **a** Cycling performance of Bi/CNRs-15//NVP at 1 A g^-1^. **b** Rate performance of Bi/CNRs-15//NVP at various current densities. **c** Charge/discharge curves of Bi/CNRs-15//NVP at various current densities. **d** Cycling performance of Bi/CNRs-15//NVP at 5 A g^-1^

Encouraged by the impressive half cell properties, a full cell was fabricated using Bi/CNRs-15 as an anode, and NVP as a cathode. The mass loadings of cathode and anode are about 3.9 and 1 mg cm^-2^, respectively, to obtain the optimal performance of the Bi/CNRs-15//NVP full cell. The capacity of the Bi/CNRs-15 anode in the half cell at the 3^th^ cycle is 393.1 mAh g^-1^ (Fig. S19b) and that of the NVP cathode is 100.1 mAh g^-1^ (Fig. S26b). The circular electrodes are discoidal pieces with a diameter of 12 mm and the corresponding area of each disk is about 1.13 cm^2^. Hence, the negative/positive (N/P) ratio = areal capacity of anode/areal capacity of cathode = 393.1 mAh g^-1^ × 1 mg cm^-2^ × 1.13 cm^2^/(100.1 mAh g^-1^ × 3.9 mg cm^-2^ × 1.13 cm^2^) = 1.01. The full cell shows a reversible capacity of 337.8 mAh g^-1^ (based on the mass of anode) after 100 cycles at 1 A g^-1^. Note that the rate performance of the full cell is also impressive with the average capacities of 372.8, 367.9, 343.1, 320.1 and 308.2 mAh g^-1^ at 0.2, 0.5, 1, 2 and 5 A g^-1^, respectively. Moreover, the full cell achieves a high capacity of 291.2 mAh g^-1^ after 400 cycles under a high current of 5 A g^-1^. All these results indicate that Bi/CNRs-15 is a feasible anode for practical applications in fast charging SIBs.


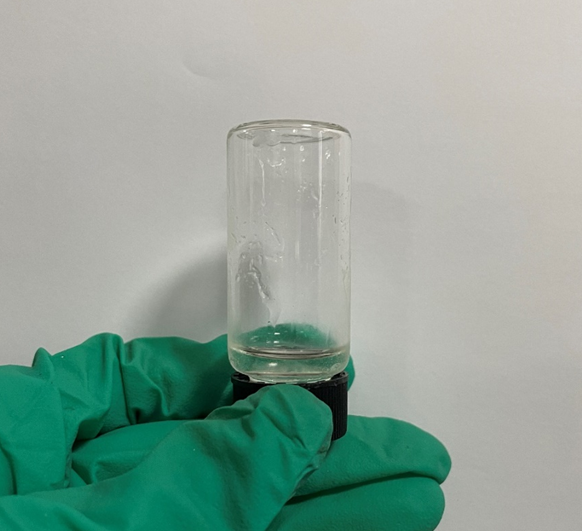


**Fig. S28** Photograph of 1.0 M NaPF_6_ in DME at -40 °C

Ether-based solvents are widely acknowledged for their low viscosity and freezing points, which enable the electrolyte to maintain a liquid state and display high ionic conductivity even at low temperature. For example, DME (1,2-dimethoxyethane) exhibits a low freezing point of -58 °C [Ref. 66 of the text]. Through further cryogenic storage experiments of electrolytes, we find that the electrolyte viscosity of 1 M NaPF_6_ in DME increases at -40 °C (Fig. S28) compared with room temperature. A small part of electrolyte hangs upside down on the wall of the glass bottle and most remains liquid. The freezing point of the electrolyte can be reduced by lowering the electrolyte concentration and/or introducing a co-solvent with a low freezing point.

**
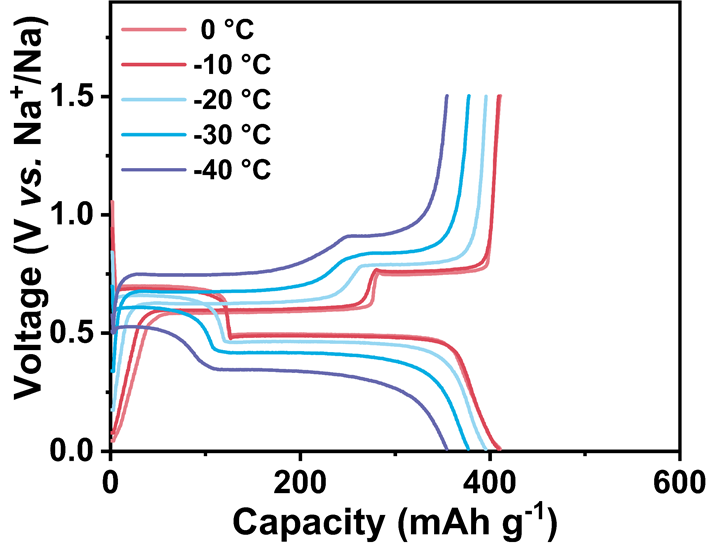
**

**Fig. S29** Charge/discharge curves of Bi/CNRs-15 at 1 A g^-1^ at different temperatures


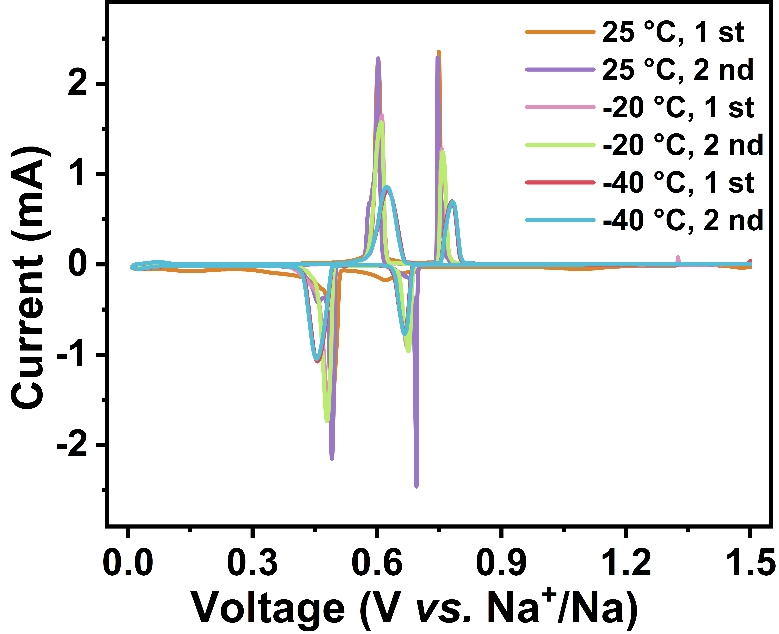


**Fig. S30** CV curves of Bi/CNRs-15 at 0.1 mV s^-1^ at different temperatures

**
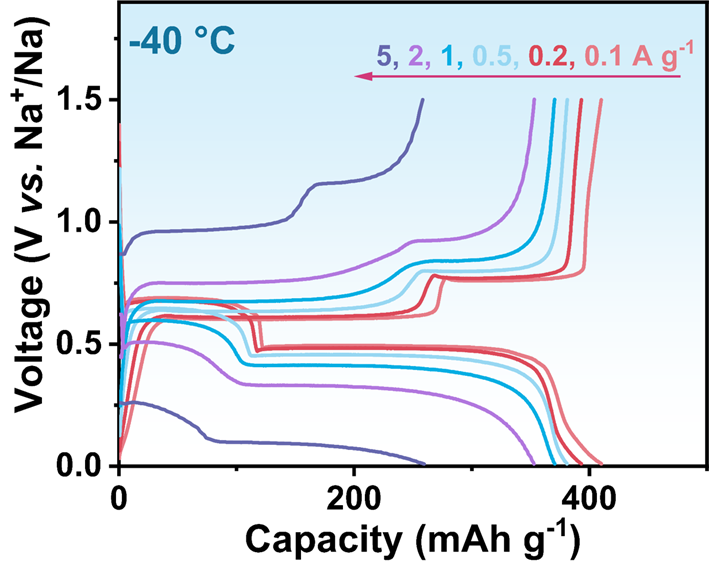
**

**Fig. S31** Charge/discharge curves of Bi/CNRs-15 at various rates at -40 °C


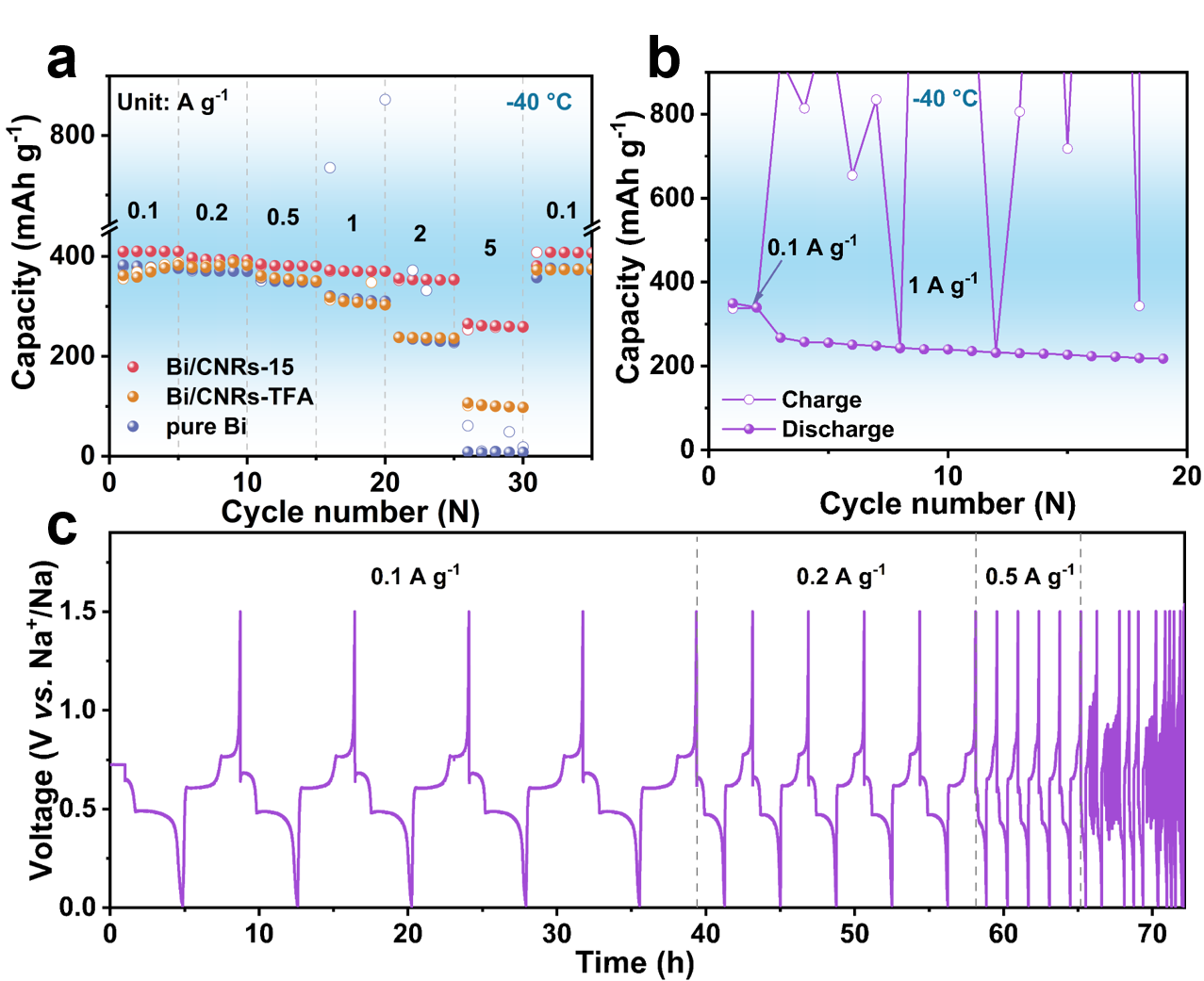


**Fig. S32** **a** Comparisons of rate performance of Bi/CNRs-15, Bi/CNRs-TFA and pure Bi at -40 °C. **b** Cycling performance of pure Bi at 1 A g^-1^ at -40 °C. **c** The voltage-time curve of pure Bi at various current densities at -40 °C.

**
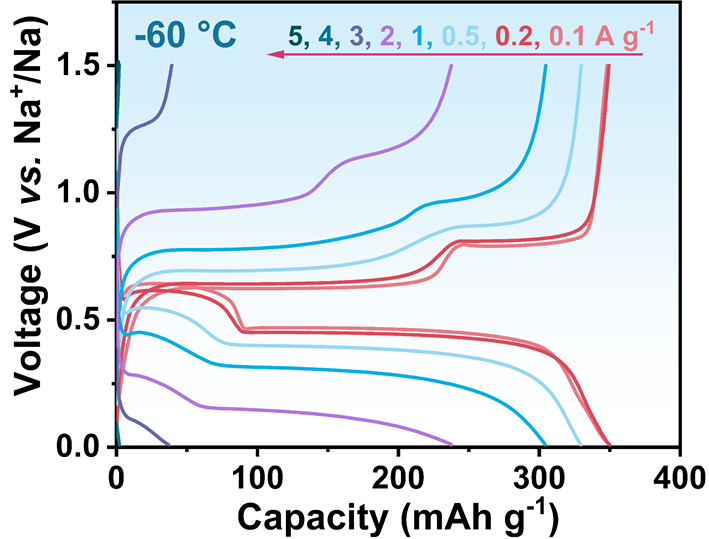
**

**Fig. S33** Charge/discharge curves of Bi/CNRs-15 at various rates at -60 °C

**
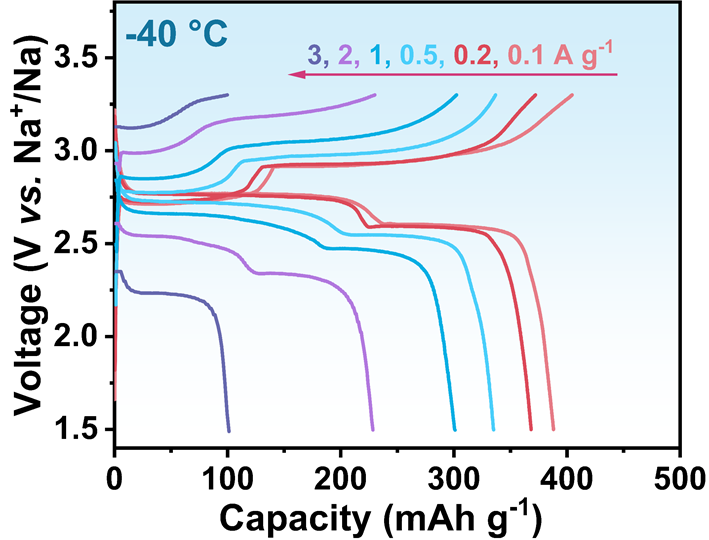
**

**Fig. S34** Charge/discharge curves of the Bi/CNRs-15//NVP full cell at various current densities at -40 °C

**
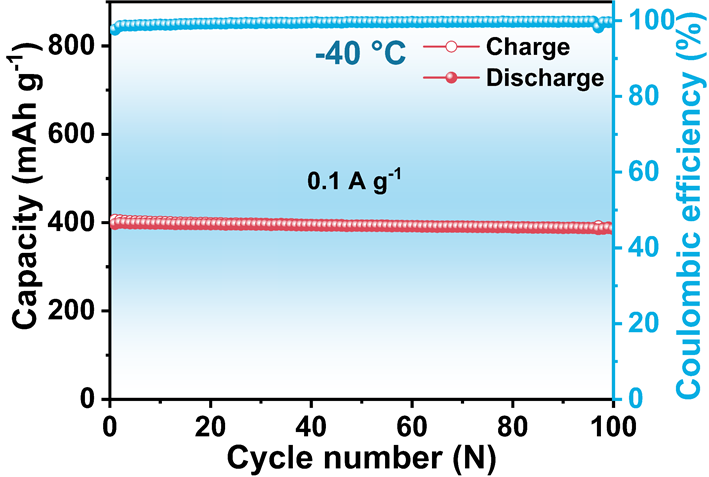
**

**Fig. S35** Cycling performance of the Bi/CNRs-15//NVP full cell after rate tests (see Fig. 2h of the text) at 0.1 A g^-1^ at -40 °C

**
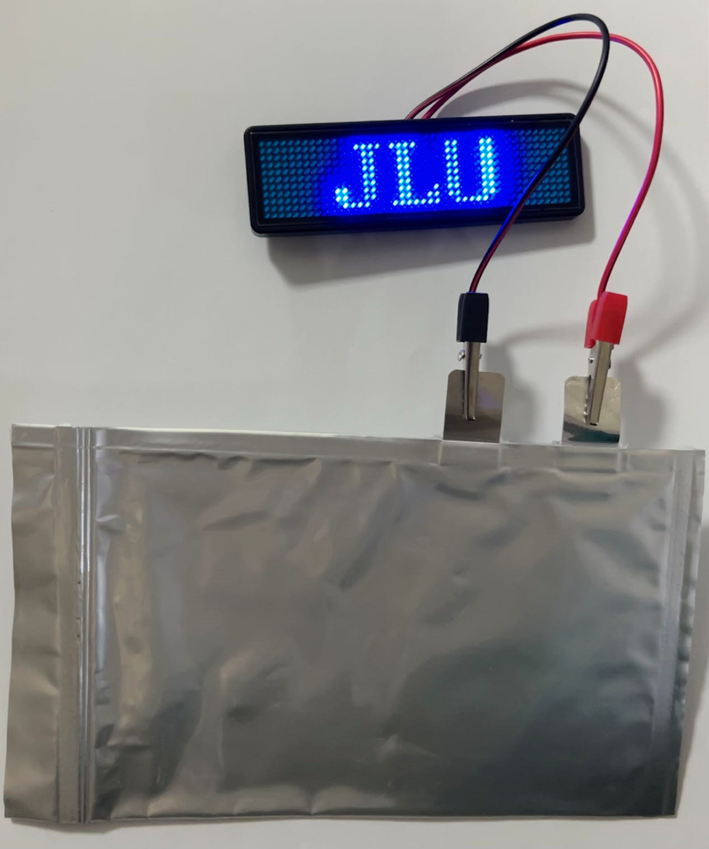
**

**Fig. S36** Photograph of lighted LED panel by the Bi/CNRs-15//NVP pouch cell at room temperature


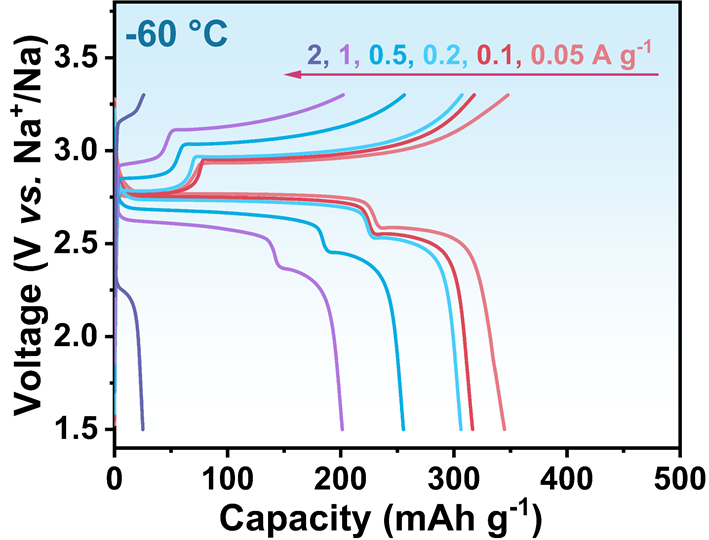


**Fig. S37** Charge/discharge curves of the Bi/CNRs-15//NVP full cell at various current densities at -60 °C


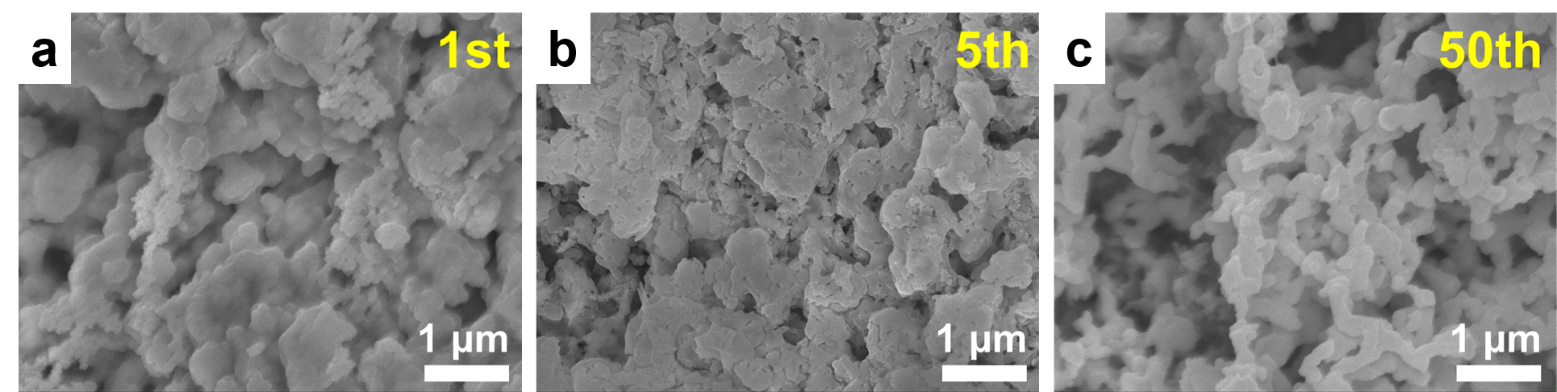


**Fig. S38 a-c** SEM images of the Bi/CNRs-15 electrode after 1, 5 and 50 cycles, respectively

The morphology of the cycled electrode was characterized by SEM and TEM. From Fig. S38, as the cycle proceeds, the electrode gradually evolves into coral-like porous nanonetworks, which can facilitate the diffusion of Na^+^ [S1, S2].


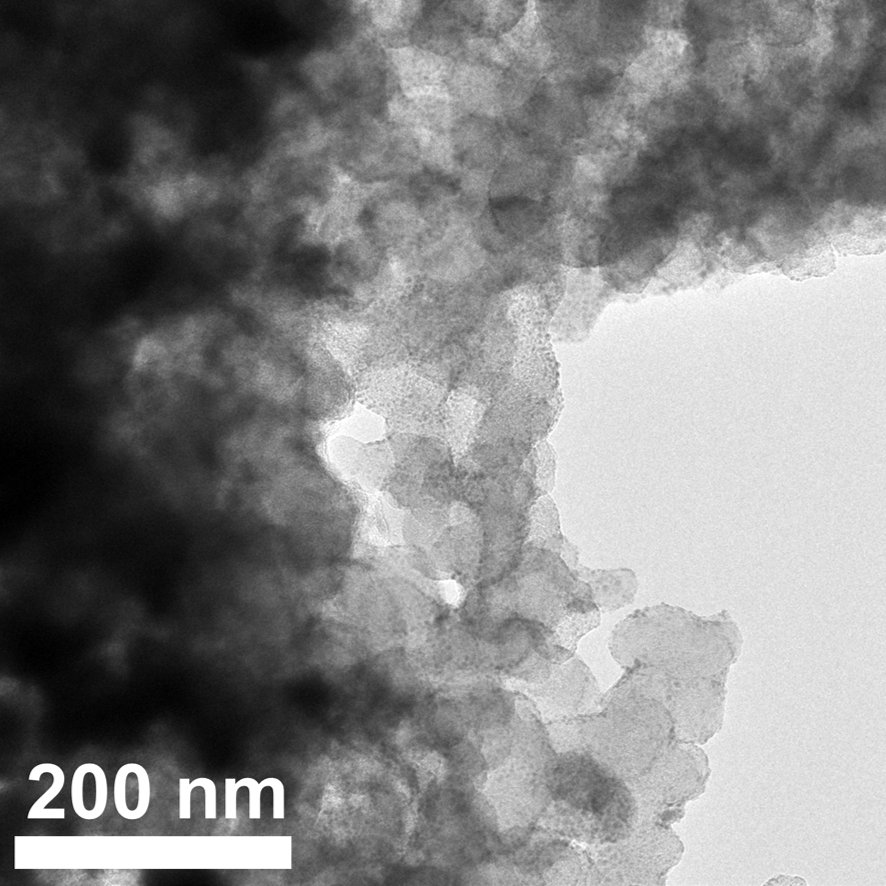


**Fig. S39** TEM image of the Bi/CNRs-15 electrode after 50 cycles

Figure S39 shows the TEM image of Bi/CNRs-15 after 50 cycles, where it can be observed that ultrasmall nanoparticles load on the porous structure.


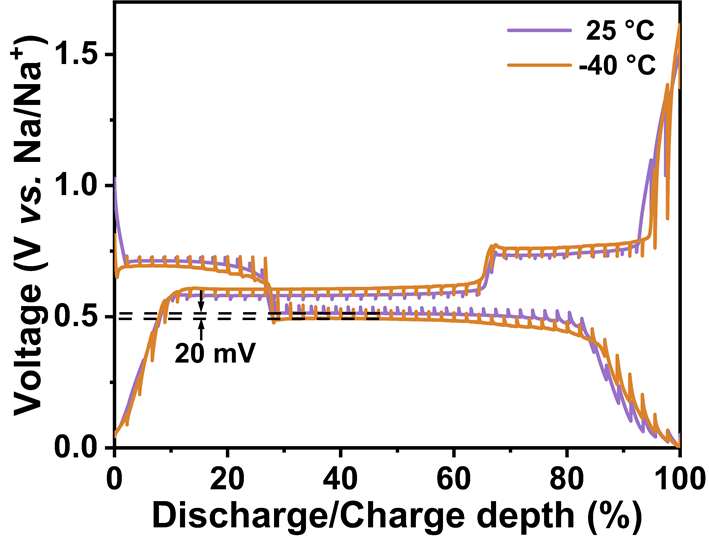


**Fig. S40** Quasi-equilibrium voltage curves of Bi/CNRs-15 from GITT at 25 and -40 °C

Fig. S40 shows a small voltage polarization (20 mV) for Bi/CNRs-15 at -40 °C, validating excellent Na^+^ transport kinetics even at low temperature. The Na^+^ diffusion coefficient (*D*) was calculated using GITT technique to expound the electrochemical reaction kinetics. The *D* value was calculated from the following equation:

*D =* $\frac{\text{4}}{\text{ π}\text{τ}\text{ }}(\frac{\text{m}_{\text{B}\text{ }}\text{V}_{\text{M}\text{ }}}{\text{M}_{\text{B}}\text{S}}$)*^2^*$(\frac{\text{ΔΕ}_{\text{s}\text{ }}}{\text{ΔΕ}_{\text{τ}\text{ }}}$)*^2^*  (S3)

where *S* is the surface area of the tested electrode and *τ* is the time duration of the pulse, *m*_B_, *M*_B_ and *V*_M_ are the weight, molar mass and molar volume of the active materials, respectively. Δ*E*_s_ is the steady-state potential change by the current pulse, and Δ*E_τ_* is the instantaneous potential change during the constant current pulse.

**
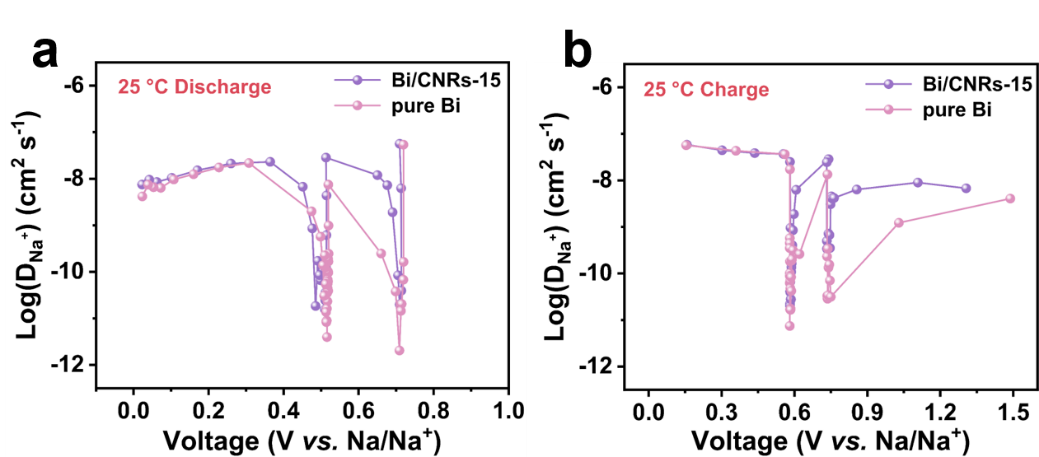
**

**Fig. S41** Na^+^ diffusion coefficients of Bi/CNRs-15 and pure Bi during the **a** discharging and **b** charging processes at 25 °C

**
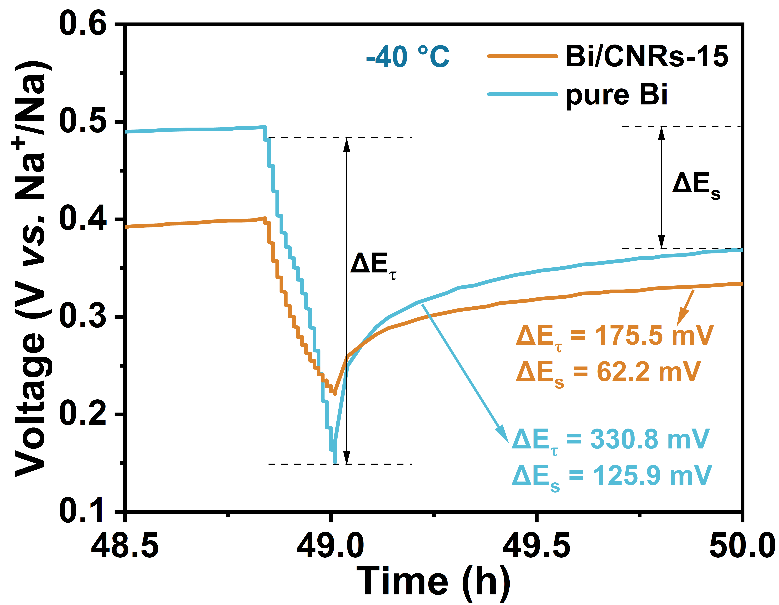
**

**Fig. S42** Detailed voltage response curves of the Bi/CNRs-15 and pure Bi electrodes during a single constant current pulse with respect to time


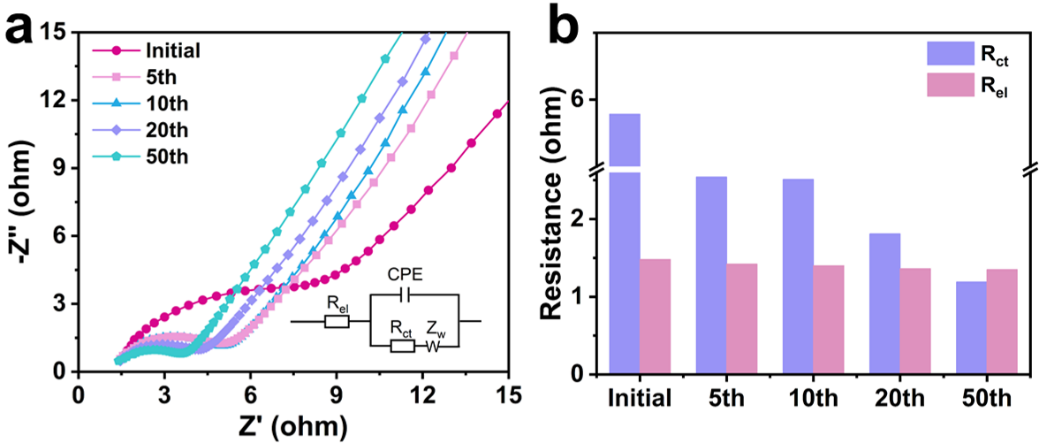


**Fig. S43 a** EIS plots and **b** corresponding *R*_el_ and *R*_ct_ values of the cycled Bi/CNR-15 electrode at room temperature

**
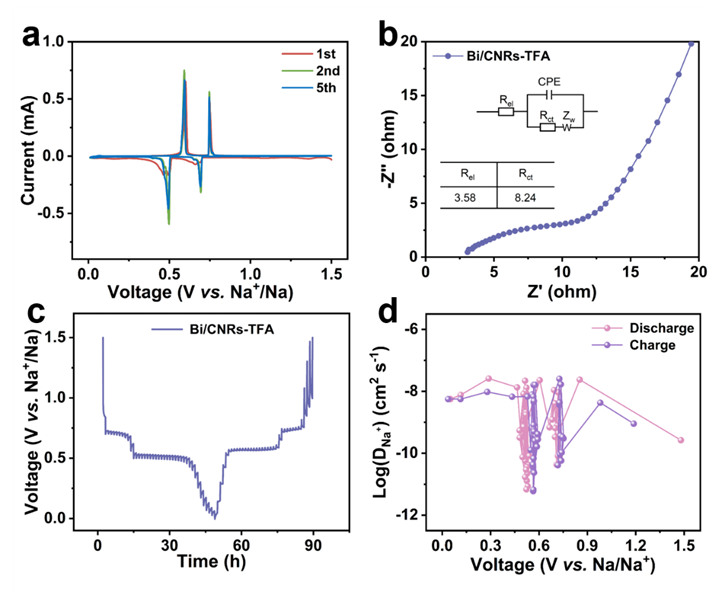
**

**Fig. S44 a** CV curves of Bi/CNRs-TFA at 0.1 mV s^-1^. **b** EIS plots of Bi/CNRs-TFA, where the inset shows corresponding equivalent circuit diagram and the values of fitted *R*_ct_ and *R*_el_. **c** GITT voltage profiles of the Bi/CNRs-TFA electrode. **d** Na^+^ diffusion coefficients of Bi/CNRs-TFA during the discharging and charging processes

Two cathodic peaks at 0.49 and 0.69 V are related to the stepwise alloy reactions from Bi to NaBi and further to Na_3_Bi (Fig. S44a). On the basis of the equivalent circuit diagram in Fig. S44b, the values of *R*_ct_ and *R*_el_ are 8.2 and 3.6 Ω, respectively, which are larger than the values of Bi/CNRs-15 (*R*_ct_ of 5.9 Ω and *R*_el_ of 1.5 Ω). Furthermore, the Na^+^ diffusion kinetic of the Bi/CNRs-TFA electrode was investigated by GITT. The Na^+^ diffusion coefficients of Bi/CNRs-TFA are in ranges of 2.6 × 10^-8^ to 6.0 × 10^-12^, which are smaller than those of Bi/CNRs-15 (5.8 × 10^-8^ to 1.6 × 10^-11^).

**
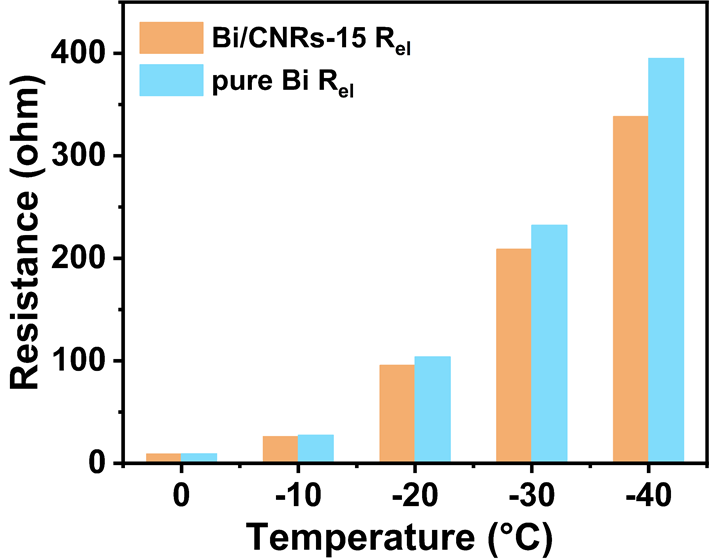
**

**Fig. S45** Comparisons of *R*_el_ of the Bi/CNRs-15 and pure Bi electrodes at various temperatures

**
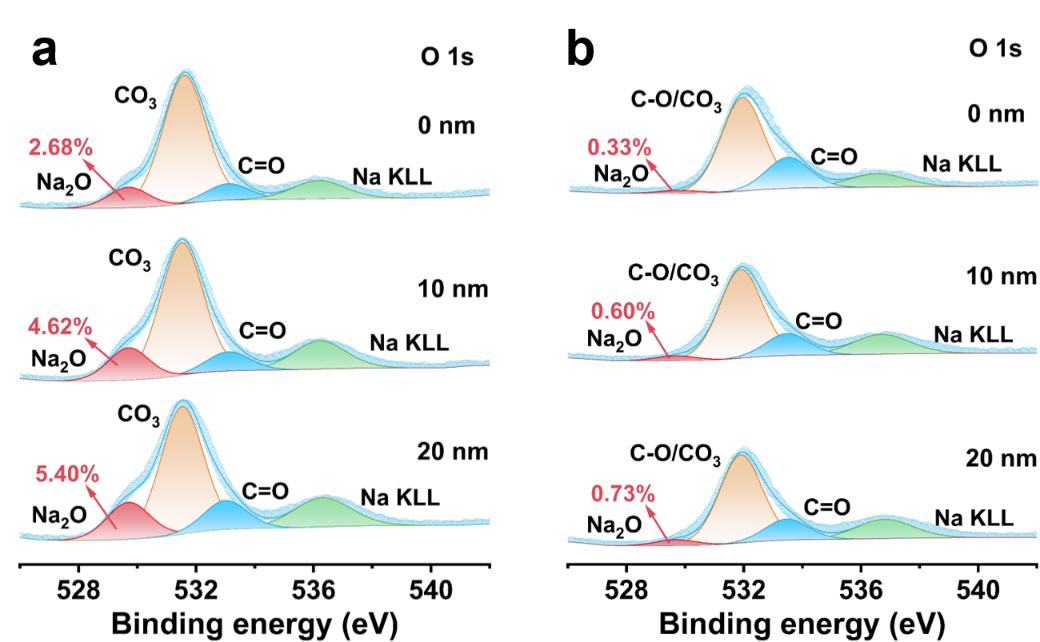
**

**Fig. S46** Depth-profiling XPS spectra of O 1s of SEI on the **a** Bi/CNRs-15 and **b** pure Bi electrodes

**
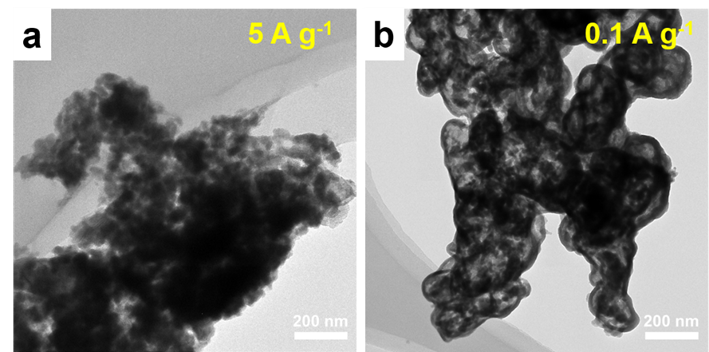
**

**Fig. S47 a** TEM image of the Bi/CNRs-15 electrode after 5 cycles at 5 A g^-1^ at -40 °C. **b** TEM image of the Bi/CNRs-15 electrode after 5 cycles at 0.1 A g^-1^ at -40 °C

**
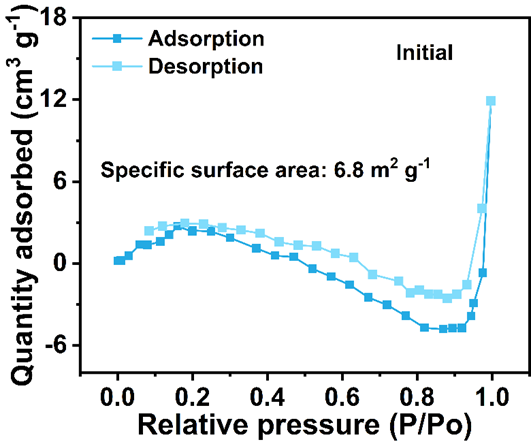
**

**Fig. S48** N_2_ adsorption/desorption isotherms of the Bi/CNRs-15 electrode before cycles

**
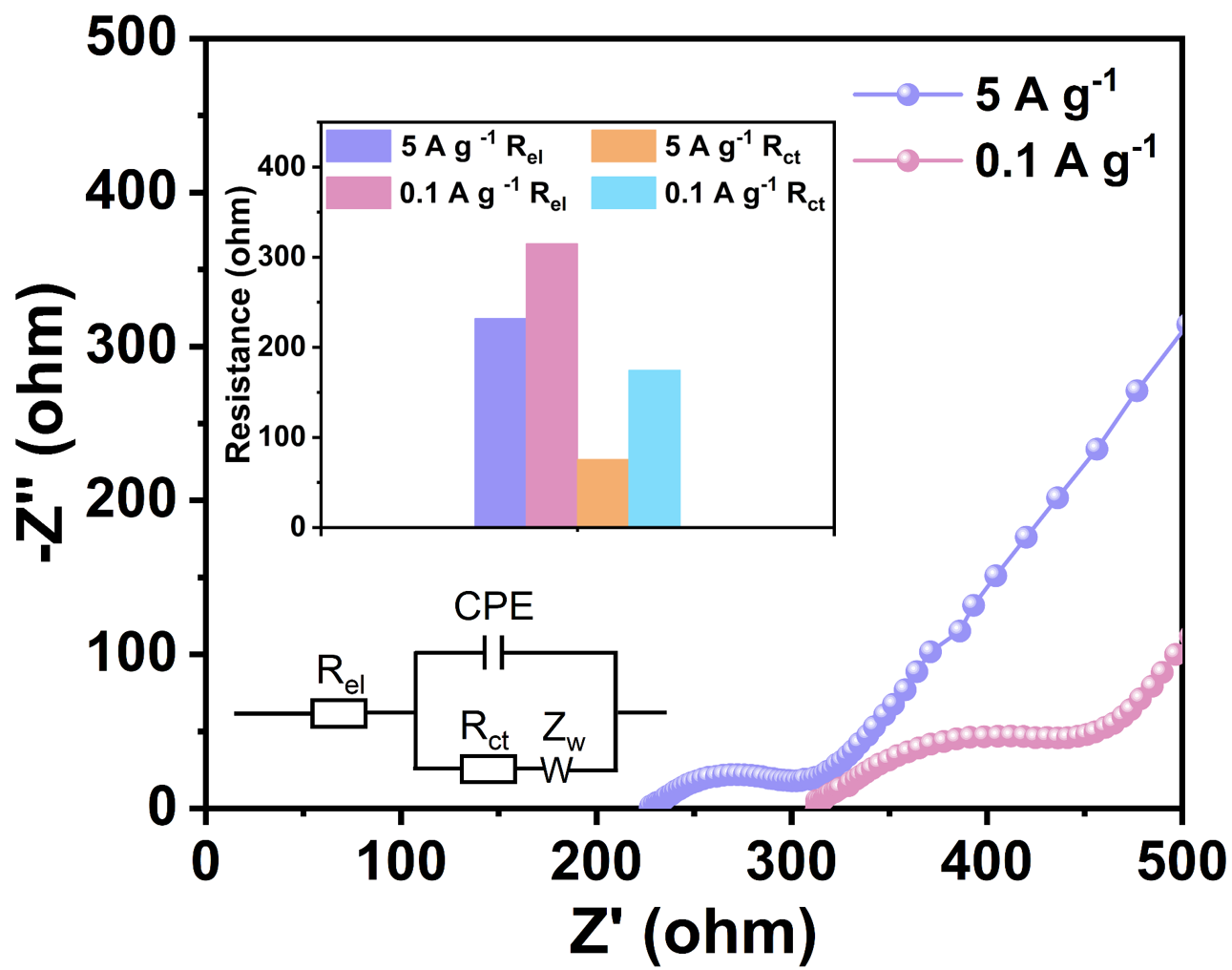
**

**Fig. S49** EIS plots of the Bi/CNRs-15 electrodes after 5 cycles at 5/0.1 A g^-1^ at -40 °C, where the inset shows corresponding equivalent circuit diagram and comparisons of *R*_ct_ and *R*_el_


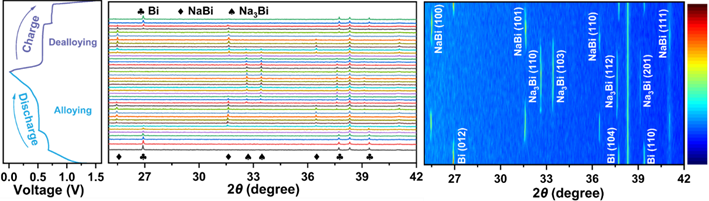


**Fig. S50** *In-situ* XRD patterns of the Bi/CNRs-15 electrode under different discharge/charge states

The *in-situ* XRD measurement was conducted to probe the Na-storage reaction mechanism of Bi/CNRs-15. The corresponding charge/discharge profile (left panel), line plot (centre panel) and contour plot (right panel) are displayed in Fig. S50. In the discharging process, the diffraction peaks of Bi at 26.9°, 37.7° and 39.4° are attenuated gradually due to alloying reaction into the NaBi phase. With continuous Na^+^ insertion, the new characteristic peaks of Na_3_Bi appear and gradually enhance while the peaks of NaBi weaken and eventually vanish, revealing that tetragonal NaBi has completely transformed into hexagonal Na_3_Bi. During the desodiation process, the peaks of NaBi arise along with the disappearing of the Na_3_Bi phase, and then all NaBi phase converts to Bi due to full charge at 1.5 V, corroborating the reversibility after alloying and dealloying reaction.

**Supplementary Tables**

**Table S1** Crystallite sizes of Bi/CNRs-15 calculated by the Scherrer equation.

| 2*θ* (°) | *β* (°) | *L* (nm) |
| --- | --- | --- |
| 27.20221 | 0.20307 | 39.80275 |
| 38.00517 | 0.22482 | 36.95764 |
| 39.66177 | 0.21785 | 38.33422 |

The average crystallite sizes of Bi nanoparticles have been calculated by the Scherrer equation *L*= Kλ/(*β*cos*θ*), where *L* is the crystallite size, K is a constant of 0.89, λ is the wavelength of the X-rays, and *β* is the half-height width of the diffraction peak.

**Table S2** Comparisons of low-temperature performances of various SIB anode materials

| Samples | Operating temperature (°C) | Rate capability (mAh g^-1^) | Cyclic stability  (mAh g^-1^) | Refs. |
| --- | --- | --- | --- | --- |
| Bi/CNRs-15 | **-40**  **-60** | **261.4 at 5 A g^-1^**  **237.9 at 2 A g^-1^** | **241.7 (2400 cycles, 1 A g^-1^)**  **334.1 (100 cycles, 0.1 A g^-1^)** | **This work** |
| CoGa_2_S_4_@G | -60 | 97 at 2 A g^-1^ | 100 (1000 cycles, 1 A g^-1^) | [S11] |
| hard carbon | -40  -50 | 255 at 0.1 A g^-1^  220 at 0.1 A g^-1^ | 243 (500 cycles, 0.1 A g^-1^)  209 (100 cycles, 0.1 A g^-1^) | [S12] |
| H-NTO | -40 | 25 at 0.3 A g^-1^ | 55 (3000 cycles, 0.1 A g^-1^) | [S13] |
| Zn-HC | -40 | 44.3 at 2 A g^-1^ | 258 (400 cycles, 0.1 A g^-1^) | [S14] |
| HCM-1300-ZBE | -40 | 295 at 0.1 A g^-1^ | 260 (70 cycles, 0.1 A g^-1^) | [S15] |
| Meso-TNOC | -40 | 59 at 1.7 A g^-1^ | 99 (500 cycles, 0.17 A g^-1^) | [S16] |
| FeSe_2_/rGO | -40 | 271.7 at 1 A g^-1^ | 216.7 (200 cycles, 1 A g^-1^) | [S17] |
| Graphite | -40 | 59 at 0.3 A g^-1^ | / | [S18] |
| Fe_3_BO_5_@C | -40 | 292 at 0.05 A g^-1^ | 350 (20 cycles, 0.1 C) | [S19] |
| VS_4_ | -40 | 163 at 2 A g^-1^ | 396 (20 cycles, 0.2 A g^-1^) | [S20] |
| Bi@C | -40 | 246 at 0.1 A g^-1^ | 246 (10 cycles, 0.1 A g^-1^) | [27 of text] |
| TiO_2_@rGO | -40  -20 | 30 at 7 A g^-1^  54 at 7 A g^-1^ | 100 (1500 cycles, 1.75 A g^-1^)  120 (1500 cycles, 1.75 A g^-1^) | [S21] |
| F-CuFeS_2_@RGO | -40  -20 | 200 at 2 A g^-1^  375 at 2 A g^-1^ | 182 (200 cycles, 2 A g^-1^)  375 (200 cycles, 2 A g^-1^) | [S22] |
| ZnSe@NCNFs | -40  -20 | /  98 at 5 A g^-1^ | 55 (1000 cycles, 1 A g^-1^)  123 (1000 cycles, 1 A g^-1^) | [S23] |
| KTOP | -35 | 75.6 at 0.45 A g^-1^ | 90 (550 cycles, 0.45 A g^-1^) | [S24] |
| TS-MoSe_2_ | -30  -10 | 308 at 0.1 A g^-1^  410 at 0.1 A g^-1^ | 260 (100 cycles, 0.1 A g^-1^) 380 (100 cycles, 0.1 A g^-1^) | [S25] |
| FePSe_3_ | -30 | 320 at 10 A g^-1^ | 200 (10000 cycles, 20 A g^-1^) | [S26] |
| PANI/Ti_3_C_2_T*_x_* | -30 | 120.5 at 0.1 A g^-1^ | 100 (100 cycles, 0.1 A g^-1^) | [S27] |
| MoS_2_@MXene@D-TiO_2_ | -30 | / | 180 (100 cycles, 0.05 A g^-1^) | [S28] |
| Ti_3_C_2_-N_funct_ | -25 | 90 at 5 A g^-1^ | 110 (5000 cycles, 1 A g^-1^) | [S29] |
| 3DSG | -25 | 84 at 2 A g^-1^ | 250 (500 cycles, 0.1 A g^-1^) | [S30] |
| defective HT-NW | -25 | 108 at 5 A g^-1^ | 120 (4200 cycles, 1 A g^-1^) | [S31] |
| c-MoS_2_ | -25 | 37 at 2 A g^-1^ | 100 (1000 cycles, 0.2 A g^-1^) | [S32] |
| FeS@g-C | -25 | / | 311 (80 cycles, 0.05 A g^-1^) | [S33] |
| Fe_7_Se_8_@C | -25 | / | 338 (80 cycles, 0.2 A g^-1^) | [S34] |
| Fe_1-_*_x_*S@NC | -25 | 159.9 at 3 A g^-1^ | / | [S35] |
| hard carbon | -20 | 175 at 2 A g^-1^ | 181 (1000 cycles, 2 A g^-1^) | [S36] |
| NaV_1.25_Ti_0.75_O_4_ | -20 | 93 at 0.2 C  1 C = 100 mA g^-1^ | / | [S37] |
| Bi@3DCF | -20 | 190 at 5 A g^-1^ | 200 (500 cycles, 1 A g^-1^) | [S38] |
| NaTi_2_(PO_4_)_3_/C foams | -20 | 95 at 20 C | 116 (500 cycles, 0.2 C) | [S39] |
| NaTi_2_(PO_4_)_3_/C-CNT | -20 | 62 at 10 C  1 C = 133 mA g^-1^ | / | [S40] |
| NTONb0.08 | -15 | 103 at 0.1 A g^-1^ | 103 (200 cycles, 0.1 A g^-1^) | [S41] |
| SnSe_2_-SePAN | -15 | 300 at 0.5 A g^-1^ | 300 (700 cycles, 0.5 A g^-1^) | [S42] |
| Sb_2_Se_3_/rGO | -15 | 233 at 2 A g^-1^ | / | [S43] |
| hard carbon  paper | -15 | 300 at 0.5 A g^-1^ | 217.1 (1000 cycles, 0.5 A g^-1^) | [S44] |
| CF | -10  0 | / | 270 (500 cycles, 0.2 A g^-1^)  227 (1000 cycles, 0.5 A g^-1^) | [S45] |
| ZnSe-40 | -10 | 181 at 5 A g^-1^ | 247 (600 cycles, 1 A g^-1^) | [S46] |
| ZnS/MWCNTs | -10 | 208 at 4 A g^-1^ | 230 (400 cycles, 1 A g^-1^) | [S47] |
| CoS/Cu_2_S@C-NC | -5 | / | 375.2 (900 cycles, 2 A g^-1^) | [S48] |
| P-NiSe@C | -5 | 343.8 at 0.2 A g^-1^ | 313.8 (50 cycles, 0.2 A g^-1^) | [S49] |
| Bi@C-NSA | 0 | 276.3 at 2 A g^-1^ | 271.8 (200 cycles, 1 A g^-1^) | [28 of text] |
| NbSSe | 0 | 85 at 3 C  1 C = 150 mAh g^-1^ | 136 (500 cycles, 0.2 C) | [S50] |
| FeS_0.5_Se_0.5_@NC | 0 | 226.1 at 8 A g^-1^ | 380.1 (200 cycles, 1 A g^-1^) | [S51] |
| Na_2_Ti_6_O_13_@C | 0 | 20 at 5 C | 43 (250 cycles, 2 C) | [S52] |
| SnSe@CNF | 0 | / | 267 (100 cycles, 0.1 A g^-1^) | [S53] |

**Supplementary References**

1. Y. Z. Liang, N. Song, Z. C. Y. Zhang, W. H. Chen, J. K. Feng et al., Integrating Bi@C nanospheres in porous hard carbon frameworks for ultrafast sodium storage. Adv. Mater. **34**, 2202673 (2022). https://doi.org/10.1002/adma.202202673
2. X. L. Cheng, R. W. Shao, D. J. Li, H. Yang, Y. Wu et al., A self-healing volume variation three-dimensional continuous bulk porous bismuth for ultrafast sodium storage. Adv. Funct. Mater. **31**, 2011264 (2021). https://doi.org/10.1002/adfm.202011264
3. X. L. Qiu, X. L. Wang, Y. X. He, J. Y. Liang, K. Liang et al., Superstructured mesocrystals through multiple inherent molecular interactions for highly reversible sodium ion batteries. Sci. Adv. **7**, eabh3482 (2021). https://doi.org/10.1126/sciadv.abh3482
4. P. X. Xiong, P. X. Bai, A. Li, B. F. Li, M. R. Cheng et al., Bismuth nanoparticle@carbon composite anodes for ultralong cycle life and high-rate sodium-ion batteries. Adv. Mater. **31**, 1904771 (2019). https://doi.org/10.1002/adma.201904771
5. H. Yang, R. Xu, Y. Yao, S. F. Ye, X. F. Zhou et al., Multicore-shell Bi@N-doped carbon nanospheres for high power density and long cycle life sodium- and potassium-ion anodes. Adv. Funct. Mater. **29**, 1809195 (2019). https://doi.org/10.1002/adfm.201809195
6. H. Yang, L. W. Chen, F. X. He, J. Q. Zhang, Y. Z. Feng et al., Optimizing the void size of yolk-shell Bi@void@C nanospheres for high-power-density sodium-ion batteries. Nano Lett. **20**, 758-767 (2020). https://doi.org/10.1021/acs.nanolett.9b04829
7. X. L. Cheng, D. J. Li, Y. Wu, R. Xu, Y. Yu, Bismuth nanospheres embedded in three-dimensional (3D) porous graphene frameworks as high performance anodes for sodium- and potassium-ion batteries. J. Mater. Chem. A **7,** 4913-4921 (2019). https://doi.org/10.1039/c8ta11947c
8. J. Chen, X. L. Fan, X. Ji, T. Gao, S. Hou et al., Intercalation of Bi nanoparticles into graphite results in an ultra-fast and ultra-stable anode material for sodium-ion batteries. Energy Environ. Sci. **11**, 1218-1225 (2018). https://doi.org/10.1039/c7ee03016a
9. S. Liu, K. Niu, S. L. Chen, X. Sun, L. H. Liu et al., TiO_2_ bunchy hierarchical structure with effective enhancement in sodium storage behaviors. Carbon Energy **4**, 645-653 (2022). https://doi.org/10.1002/cey2.172
10. H. C. Jin, S. Xin, C. H. Chuang, W. D. Li, H. Y. Wang et al., Black phosphorus composites with engineered interfaces for high-rate high-capacity lithium storage. Science **370**, 192-197 (2020). https://doi.org/10.1126/science.aav5842
11. F. J. Mo, Z. X. Lian, B. W. Fu, Y. Song, P. Wang et al., A novel composite strategy to build a sub-zero temperature stable anode for sodium-ion batteries. J. Mater. Chem. A **7**, 9051-9058 (2019). https://doi.org/10.1039/C9TA02067E
12. C. Yang, X. W. Liu, Y. Lin, L. M. Yin, J. Lu et al., Entropy-driven solvation toward low-temperature sodium-ion batteries with temperature-adaptive feature. Adv. Mater. **35**, 2301817 (2023). https://doi.org/10.1002/adma.202301817
13. W. J. Meng, Z. Z. Dang, D. S. Li, L. Jiang, Long-cycle-life sodium-ion battery fabrication via a unique chemical bonding interface mechanism. Adv. Mater. **35**, 2301376 (2023). https://doi.org/10.1002/adma.202301376
14. Z. X. Lu, J. Wang, W. L. Feng, X. P. Yin, X. C. Feng et al., Zinc single-atom-regulated hard carbons for high-rate and low-temperature sodium-ion batteries. Adv. Mater. **35**, 2211461 (2023). https://doi.org/10.1002/adma.202211461
15. X. P. Yin, Z. X. Lu, J. Wang, X. C. Feng, S. Roy et al., Enabling fast Na^+^ transfer kinetics in the whole-voltage-region of hard-carbon anodes for ultrahigh-rate sodium storage. Adv. Mater. **34**, 2109282 (2022). https://doi.org/10.1002/adma.202109282
16. H. C. Liang, L. L. Liu, N. Wang, W. Zhang, C. T. Hung et al., Unusual mesoporous titanium niobium oxides realizing sodium-ion batteries operated at -40 °C. Adv. Mater. **34**, 2202873 (2022). https://doi.org/10.1002/adma.202202873
17. Y. Tian, J. G. Lu, H. C. Tang, X. Wang, L. Q. Zhang et al., An ultra-stable anode material for high/low-temperature workable super-fast charging sodium-ion batteries. Chem. Eng. J. **422**, 130054 (2021). https://doi.org/10.1016/j.cej.2021.130054
18. J. W. Chen, Y. Peng, Y. Yin, Z. Fang, Y. J. Cao et al., A desolvation-free sodium dual-ion chemistry for high power density and extremely low temperature. Angew. Chem. Int. Ed. **60**, 23858-23862 (2021). https://doi.org/10.1002/anie.202110501
19. Y. J. Cao, X. L. Cao, X. L. Dong, X. Zhang, J. Xu et al., All-climate iron-based sodium-ion full cell for energy storage. Adv. Funct. Mater. **31**, 2102856 (2021). https://doi.org/10.1002/adfm.202102856
20. D. Yang, S. P. Zhang, P. Yu, S. L. Cheng, Z. S. Yuan et al., Structure engineering of vanadium tetrasulfides for high-capacity and high-rate sodium storage. Small **18**, 2107058 (2022). https://doi.org/10.1002/smll.202107058
21. D. R. Deng, X. Y. Cui, Q. H. Wu, M. S. Zheng, Q. F. Dong, In-situ synthesis TiO_2_ nanosheets@rGO for ultrafast sodium ion storage at both room and low temperatures. J. Alloy. Compound. **835**, 155413 (2020). https://doi.org/10.1016/j.jallcom.2020.155413
22. G. Sun, H. Z. Lin, R. Y. Tian, Z. X. Wei, X. Q. Wang et al., Rational design and synthesis of nanosheets self-assembled hierarchical flower-ball-like CuFeS_2_ for boosted wide temperature sodium-ion batteries. Nano Res. **16**, 9407-9415 (2023). https://doi.org/10.1007/s12274-023-5614-1
23. X. W. Wang, W. M. Zhao, W. Zhang, K. W. Wong, J. W. Wu et al., Ultrafine ZnSe encapsulated in nitrogen-doped porous carbon nanofibers for superior Na-ion batteries with a long lifespan and low-temperature performance. ACS Sustainable Chem. Eng. **9**, 11705-11713 (2021). https://doi.org/10.1021/acssuschemeng.1c02447
24. Y. R. Qi, J. Li, W. Zhong, S. J. Bao, M. W. Xu, KTiOPO_4_: A long-life, high-rate and low-temperature-workable host for Na/K-ion batteries. Chem. Eng. J. **417**, 128159 (2021). https://doi.org/10.1016/j.cej.2020.128159
25. M. X. Jiang, Y. J. Hu, B. G. Mao, Y. X. Wang, Z. Yang et al., Strain-regulated Gibbs free energy enables reversible redox chemistry of chalcogenides for sodium ion batteries. Nat. Commun. **13**, 5588 (2022). https://doi.org/10.1038/s41467-022-33329-2
26. S. H. Yuan, W. Q. Zhao, Z. H. Zeng, Y. Dong, F. Jiang et al., Triggering highly conductive FePSe_3_ with Cu-based coordination towards all-climate ultrafast sodium ion storage. J. Mater. Chem. A **10**, 22645-22661 (2022). https://doi.org/10.1039/D2TA04174J
27. X. Wang, J. Wang, J. W. Qin, X. Xie, R. Yang et al., Surface charge engineering for covalently assembling three-dimensional MXene network for all-climate sodium ion batteries. ACS Appl. Mater. Interfaces **12**, 39181-39194 (2020). https://doi.org/10.1021/acsami.0c10605
28. H. W. Zhang, J. J. Song, J. Y. Li, J. N. Feng, Y. Y. Ma et al., Interlayer-expanded MoS_2_ nanoflowers vertically aligned on MXene@dual-phased TiO_2_ as high-performance anode for sodium-ion batteries. ACS Appl. Mater. Interfaces **14**, 16300-16309 (2022). https://doi.org/10.1021/acsami.2c02080
29. Y. Xia, L. F. Que, F. D. Yu, L. Deng, Z. J. Liang et al., Tailoring nitrogen terminals on MXene enables fast charging and stable cycling Na-ion batteries at low temperature. Nano-Micro Lett. **14**, 143 (2022). https://doi.org/10.1007/s40820-022-00885-7
30. Y. Y. Wang, B. H. Hou, J. Z. Guo, Q. L. Ning, W. L. Pang et al., An ultralong lifespan and low-temperature workable sodium-ion full battery for stationary energy storage. Adv. Energy Mater. **8**, 1703252 (2018). https://doi.org/10.1002/aenm.201703252
31. M. Y. Sun, F. D. Yu, Y. Xia, L. Deng, Y. S. Jiang et al., Trigger Na^+^-solvent co-intercalation to achieve high-performance sodium-ion batteries at subzero temperature. Chem. Eng. J. **430**, 132750 (2022). https://doi.org/10.1016/j.cej.2021.132750
32. J. Lin, Y. H. Shi, Y. F. Li, X. L. Wu, J. P. Zhang et al., Confined MoS_2_ growth in a unique composite matrix for ultra-stable and high-rate lithium/sodium-ion anodes. Chem. Eng. J. **428**, 131103 (2022). https://doi.org/10.1016/j.cej.2021.131103
33. H. H. Fan, H. H. Li, J. Z. Guo, Y. P. Zheng, K. C. Huang et al., Target construction of ultrathin graphitic carbon encapsulated FeS hierarchical microspheres featuring superior low-temperature lithium/sodium storage properties. J. Mater. Chem. A **6**, 7997-8005 (2018). https://doi.org/10.1039/c8ta01392f
34. H. H. Fan, H. H. Li, Z. W. Wang, W. L. Li, J. Z. Guo et al., Tailoring coral-like Fe_7_Se_8_@C for superior low-temperature Li/Na-ion half/full batteries: synthesis, structure, and DFT studies. ACS Appl. Mater. Interfaces **11**, 47886-47893 (2019). https://doi.org/10.1021/acsami.9b15765
35. H. H. Fan, B. W. Qin, Z. W. Wang, H. H. Li, J. Z. Guo et al., Pseudocapacitive sodium storage of Fe_1-x_S@N-doped carbon for low-temperature operation. Sci. China Mater. **63**, 505-515 (2020). https://doi.org/10.1007/s40843-019-1220-2
36. Z. Tang, H. Wang, P. F. Wu, S. Y. Zhou, Y. C. Huang et al., Electrode-electrolyte interfacial chemistry modulation for ultra-high rate sodium-ion batteries. Angew. Chem. Int. Ed. **61**, e202200475 (2022). https://doi.org/10.1002/anie.202200475
37. Q. Li, K. Z. Jiang, X. Li, Y. Qiao, X. Y. Zhang et al., A high-crystalline NaV_1.25_Ti_0.75_O_4_ anode for wide-temperature sodium-ion battery. Adv. Energy Mater. **8**, 1801162 (2018). https://doi.org/10.1002/aenm.201801162
38. B. Wang, H. Yang, Y. Feng, S. Zeng, L. Tan et al., Boosting low-temperature sodium/potassium storage performance of Bi via novel electrochemical milling process. Mater. Today Energy **20**, 100627 (2021). https://doi.org/10.1016/j.mtener.2020.100627
39. X. H. Rui, X. H. Zhang, S. T. Xu, H. T. Tan, Y. Jiang et al., A low-temperature sodium-ion full battery: superb kinetics and cycling stability. Adv. Funct. Mater. **31**, 2009458 (2021). https://doi.org/10.1002/adfm.202009458
40. L. Wang, B. Wang, G. J. Liu, T. F. Liu, T. T. Gao et al., Carbon nanotube decorated NaTi_2_(PO_4_)_3_/C nanocomposite for a high-rate and low-temperature sodium-ion battery anode. RSC Adv. **6**, 70277-70283 (2016). https://doi.org/10.1039/C6RA11042H
41. C. Y. Hu, Y. Li, D. Wang, C. J. Wu, F. Chen et al., Improving low-temperature performance and stability of Na_2_Ti_6_O_13_ anodes by the Ti-O spring effect through Nb-doping. Angew. Chem. Int. Ed. **62**, e202312310 (2023). https://doi.org/10.1002/anie.202312310
42. Y. Y. Wang, F. Y. Xiao, X. Chen, P. X. Xiong, C. Y. Lin et al., Extraordinarily stable and wide-temperature range sodium/potassium-ion batteries based on 1D SnSe_2_-SePAN composite nanofibers. InfoMat **5**, e12467 (2023). https://doi.org/10.1002/inf2.12467
43. L. L. Hu, J. Pan, P. Zhao, G. J. Shi, B. F. Wang et al., A new method of synthesis of Sb_2_Se_3_/rGO as a high-rate and low-temperature anode for sodium-ion batteries. Mater. Adv. **3**, 3554-3561 (2022). https://doi.org/10.1039/D2MA00170E
44. B. H. Hou, Y. Y. Wang, Q. L. Ning, W. H. Li, X. T. Xi et al., Self-supporting, flexible, additive-free, and scalable hard carbon paper self-interwoven by 1D microbelts: superb room/low-temperature sodium storage and working mechanism. Adv. Mater. **31**, 1903125 (2019). https://doi.org/10.1002/adma.201903125
45. L. Tao, P. Sittisomwong, B. Y. Ma, A. Y. Hu, D. W. Xia et al., Tailoring solid-electrolyte interphase and solvation structure for subzero temperature, fast-charging, and long-cycle-life sodium-ion batteries. Energy Storage Mater. **55**, 826-835 (2023). https://doi.org/10.1016/j.ensm.2022.12.042
46. Y. M. Zhou, X. H. Sun, A. R. Fan, Y. P. Shang, K. Z. Xiong et al., ZnSe nanoparticles combined with uniform 3D interconnected MWCNTs conductive network as high-rate and freeze-resistant anode materials for sodium-ion batteries. Appl. Surf. Sci. **538**, 148194 (2021). https://doi.org/10.1016/j.apsusc.2020.148194
47. A. R. Fan, T. Y. Hou, X. H. Sun, D. L. Xie, X. Li et al., One-pot hydrothermal synthesis of ZnS nanospheres anchored on 3D conductive MWCNTs networks as high-rate and cold-resistant anode materials for sodium-ion batteries. ChemElectroChem **7**, 1904-1913 (2020). https://doi.org/10.1002/celc.202000204
48. X. F. Huang, K. H. Tao, T. L. Han, J. J. Li, H. G. Zhang et al., Long-cycling-life sodium-ion battery using binary metal sulfide hybrid nanocages as anode. Small **19**, 2302706 (2023). https://doi.org/10.1002/smll.202302706
49. C. Q. Su, Q. Ru, S. K. Cheng, Y. Q. Gao, F. M. Chen et al., 3D pollen-scaffolded NiSe composite encapsulated by MOF-derived carbon shell as a high-low temperature anode for Na-ion storage. Compos. Part B Eng. **179**, 107538 (2019). https://doi.org/10.1016/j.compositesb.2019.107538
50. L. F. Zhou, X. W. Gao, T. Du, H. Gong, L. Y. Liu et al., Two-dimensional NbSSe as anode material for low-temperature sodium-ion batteries. Chem. Eng. J. **435**, 134838 (2022). https://doi.org/10.1016/j.cej.2022.134838
51. Y. F. Wang, Y. Liu, Q. M. Li, Z. Q. Li, A. Xu et al., New dual-anions FeS_0.5_Se_0.5_@NC porous nanorods as advanced electrode materials for wide-temperature sodium-ion half/full batteries. Appl. Surf. Sci. **620**, 156836 (2023). https://doi.org/10.1016/j.apsusc.2023.156836
52. Q. S. Lai, J. J. Mu, Z. M. Liu, L. K. Zhao, X. W. Gao et al., Tunnel-type Na_2_Ti_6_O_13_@Carbon nanowires as anode materials for low-temperature sodium-ion batteries. Batteries Supercaps **6**, e202200549 (2023). https://doi.org/10.1002/batt.202200549
53. X. Q. Zhou, S. S. Ding, H. C. He, Z. Huang, M. Q. Cai et al., Encapsulated SnSe in carbon nanofibers as anode of sodium ion batteries with improved properties. Ionics **26**, 3937-3946 (2020). https://doi.org/10.1007/s11581-019-03382-x
